# A novel peptide encoded by N6-methyladenosine modified circMAP3K4 prevents apoptosis in hepatocellular carcinoma

**DOI:** 10.1186/s12943-022-01537-5

**Published:** 2022-04-02

**Authors:** Jin-Ling Duan, Wei Chen, Juan-Juan Xie, Mao-Lei Zhang, Run-Cong Nie, Hu Liang, Jie Mei, Kai Han, Zhi-Cheng Xiang, Feng-Wei Wang, Kai Teng, Ri-Xin Chen, Min-Hua Deng, Yi-Xin Yin, Nu Zhang, Dan Xie, Mu-Yan Cai

**Affiliations:** 1 State Key Laboratory of Oncology in South China, Collaborative Innovation Center for Cancer Medicine, Sun Yat-Sen University Cancer Center, Guangzhou, 510060 Guangdong China; 2 Department of Pathology, Sun Yat-Sen University Cancer Center, Guangzhou, 510060 Guangdong China; 3 Center of Hepato-Pancreatico-Biliary Surgery, The First Affiliated Hospital of Sun Yat-Sen University, Guangzhou, 510080 Guangdong China; 4 Department of Neurosurgery, The First Affiliated Hospital of Sun Yat-sen University, Guangdong Provincial Key Laboratory of Brain Function and Disease, Guangdong Translational Medicine Innovation Platform, Guangzhou, Guangdong 510080 China; 5 Department of Surgery, Sun Yat-Sen University Cancer Center, Guangzhou, 510060 Guangdong China; 6 Department of Nasopharyngeal Carcinoma, Sun Yat-Sen University Cancer Center, Guangzhou, 510060 Guangdong China; 7 Department of Radiation Oncology, Shandong Cancer Hospital and Institute, Shandong First Medical University and Shandong Academy of Medical Sciences, Jinan, 250117 Shandong China; 8 Department of Thoracic Surgery, Guangdong Provincial People’s Hospital, Research Center of Medical Sciences, Guangdong Academy of Medical Sciences, Guangdong Provincial People’s Hospital, Guangdong Academy of Medical Sciences, Guangzhou, 510080 Guangdong China

**Keywords:** Hepatocellular carcinoma, circMAP3K4, N6-methyadenosine, Translation, AIF, MIB1

## Abstract

**Background:**

Circular RNAs (circRNAs) regulate various biological activities and have been shown to play crucial roles in hepatocellular carcinoma (HCC) progression. However, only a few coding circRNAs have been identified in cancers, and their roles in HCC remain elusive. This study aimed to identify coding circRNAs and explore their function in HCC.

**Methods:**

CircMAP3K4 was selected from the CIRCpedia database. We performed a series of experiments to determine the characteristics and coding capacity of circMAP3K4. We then used *in vivo* and *in vitro* assays to investigate the biological function and mechanism of circMAP3K4 and its protein product, circMAP3K4-455aa, in HCC.

**Results:**

We found circMAP3K4 to be an upregulated circRNA with coding potential in HCC. IGF2BP1 recognized the circMAP3K4 N6-methyladenosine modification and promoted its translation into circMAP3K4-455aa. Functionally, circMAP3K4-455aa prevented cisplatin-induced apoptosis in HCC cells by interacting with AIF, thus protecting AIF from cleavage and decreasing its nuclear distribution. Moreover, circMAP3K4-455aa was degraded through the ubiquitin–proteasome E3 ligase MIB1 pathway. Clinically, a high level of circMAP3K4 is an independent prognostic factor for adverse overall survival and adverse disease-free survival of HCC patients.

**Conclusions:**

CircMAP3K4 is a highly expressed circRNA in HCC. Driven by m6A modification, circMAP3K4 encoded circMAP3K4-455aa, protected HCC cells from cisplatin exposure, and predicted worse prognosis of HCC patients. Targeting circMAP3K4-455aa may provide a new therapeutic strategy for HCC patients, especially for those with chemoresistance.

**Graphical Abstract:**

CircMAP3K4 is a highly expressed circRNA in HCC. Driven by m6A modification, IGF2BP1 facilitates circMAP3K4 peptide translation, then the circMAP3K4 peptide inhibits AIF cleavage and nuclear distribution, preventing HCC cells from cell death under stress and promoting HCC progression.

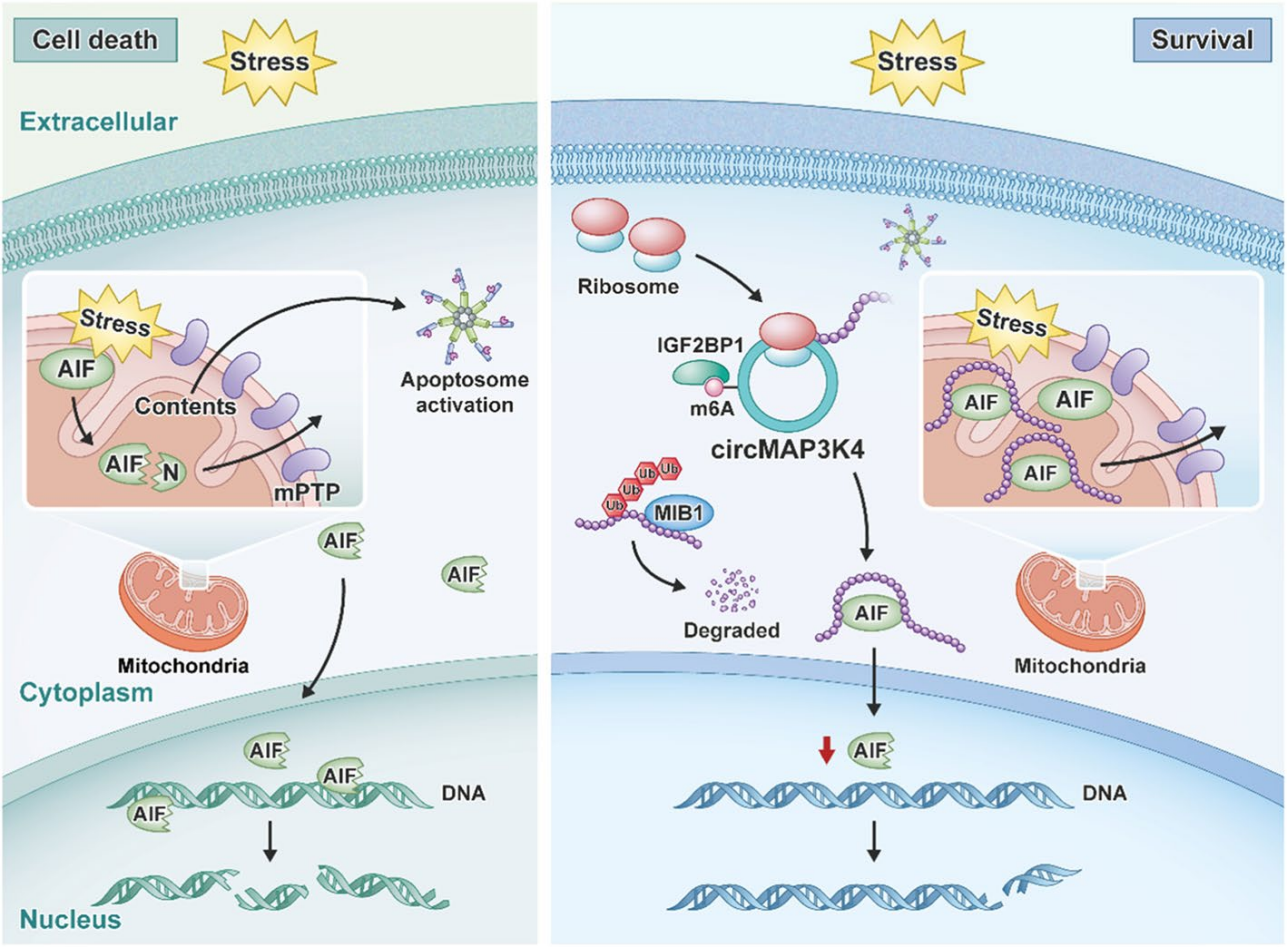

**Supplementary Information:**

The online version contains supplementary material available at 10.1186/s12943-022-01537-5.

## Introduction

Hepatocellular carcinoma (HCC) is one of the most common malignancies and a leading cause of cancer-related deaths worldwide [1]. Advances in diagnostic technologies have enabled early detection of HCCs such that they are amenable to radical local treatments including resection, liver transplantation, or local ablation [2]. However, most patients present at more developed stages, at which point combination therapies with tyrosine kinase inhibitors (TKIs) and immune checkpoint blockade have shown some promise in 14% to 36% of HCC but are still under evaluation in large-scale clinical trials [3]. Despite these novel treatments, systemic chemotherapy, including cisplatin, doxorubicin, or 5-fluorouracil, remains an effective approach for patients with advanced stage HCC or recurrence after standard treatment [4–6]. Chemoresistance is a major obstacle to obtaining satisfactory clinical responses in HCC, but the underlying mechanisms responsible for this are not well understood.

Genetic alterations have been shown to drive tumorigenesis and progression of HCC, but mutations alone can’t fully explain the resistance to apoptosis induced by chemotherapy [7]. Recent findings have demonstrated that non-coding RNAs are systematically altered in HCC. Circular RNAs (circRNAs) are a type of non-coding RNA with covalently single-stranded loops generated through back-splicing [8]. Numerous studies have suggested that circRNAs play important roles in various human tumors, including HCC, by acting as microRNA (miRNA) sponges or as scaffolds for RNA-binding proteins [9–13]. Recently, several circRNAs, including cSMARCA5, circRNA-SORE, circUHRF1, and circRNA MAT2B, have been shown to be involved in the growth, migration, metabolism, or microenvironment of HCC [14–18]. However, only a minor fraction of circRNAs have been investigated in HCC to date, and the role of coding circRNAs in HCC has not been fully elucidated.

CircRNAs were previously considered to be non-coding RNAs because they lack traditional essential elements for cap-dependent translation, such as a 5’ cap and poly (A) tail [19]. However, increasing evidence has demonstrated that some circRNAs could encode for peptides through cap-independent translation mechanisms, including internal ribosome entry sites (IRESs) and N6-methyladenosine (m6A) modification [19]. So far, a small subset of the translated circRNAs, including circ-ZNF609, circMbl, circFBXW7, circPINTexon2, and circ-SHPRH, engage in the IRES-mediated approach [20–23]. Under specific conditions, very limited circRNAs can be translated into peptides in a m6A-dependent process [24]. Additional efforts are needed to address the role of m6A modification in the biogenesis and function of circRNAs.

In this study, we demonstrate the coding ability of circMAP3K4, a novel circRNA overexpressed in HCC. CircMAP3K4 translation produced a 455 amino acid (aa) protein, circMAP3K4-455aa, facilitated by IGF2BP1 through m6A modification recognition. CircMAP3K4-455aa prevents HCC cells from cisplatin-induced apoptosis by interacting with apoptosis inducing factor mitochondria associated 1 (AIF). Our findings highlight the role of m6A modification in circRNA translation and suggest that circMAP3K4 at least partly contributes to cisplatin chemoresistance and could be a potential therapeutic target in HCC.

## Materials and methods

Detailed protocols are provided in the supplementary information.

## Results

### Selection of translated and highly expressed circRNAs in HCC

**Fig. 1:**
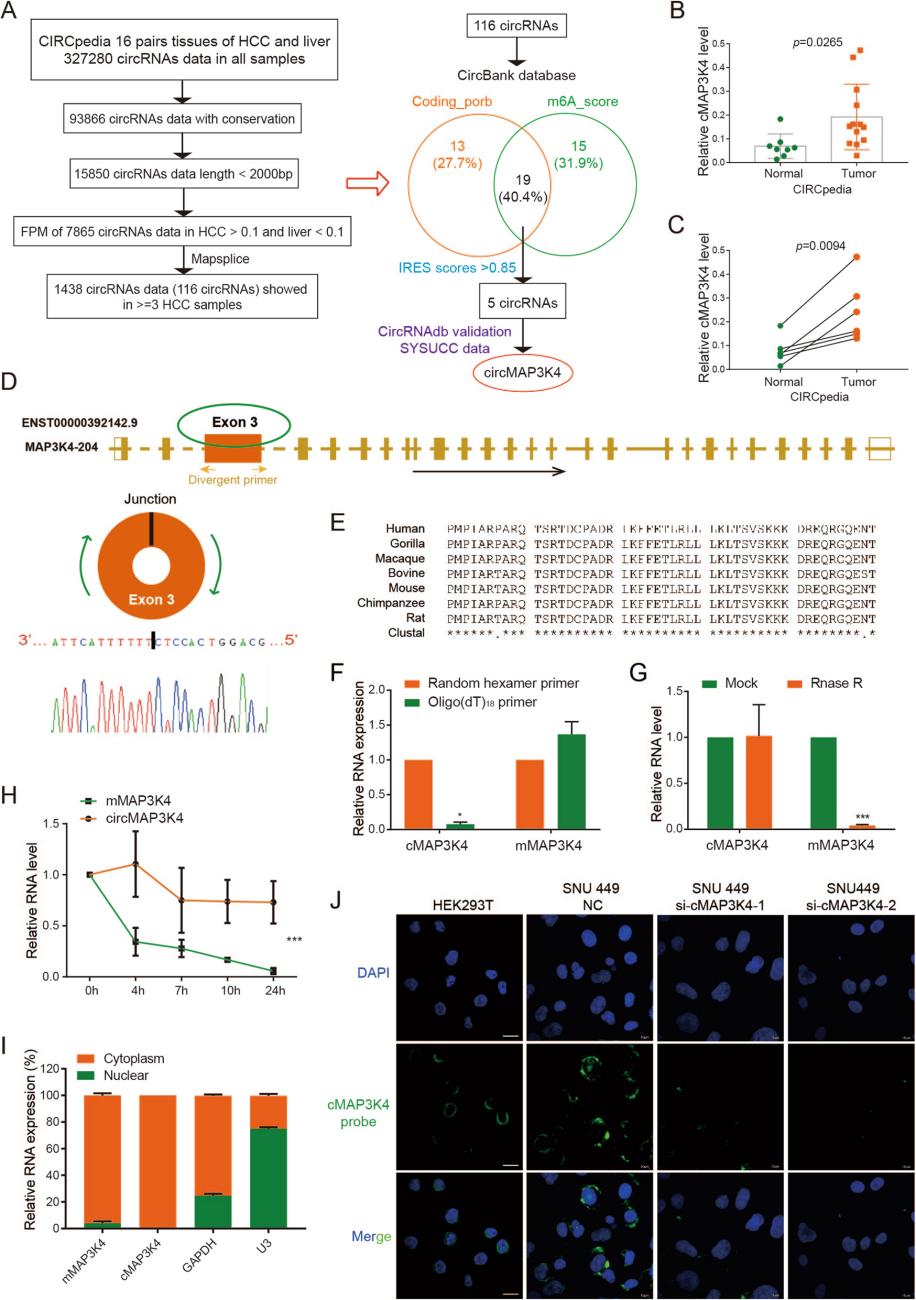
Selection and validation of circMAP3K4**. A** Flowcharts showing the selection of circRNAs overexpressed in HCC with coding potential. **B** CircMAP3K4 expression in HCC and liver tissues from the CIRCpedia database. Tumor samples, *n* = 13; liver samples, *n* = 8. **C** CircMAP3K4 expression in paired HCC and adjacent liver tissues from the CIRCpedia database (*n* = 6). **D** The location of circMAP3K4 in the human genome (upper panel), and circMAP3K4 sketch map. CircMAP3K4 expression detected by RT-qPCR followed by Sanger sequencing using divergent primers. **E** CircMAP3K4 interspecies sequence conservation. Homo sapiens, Human; Gorilla gorilla, Gorilla; Macaca mulatta, Macaque; Bovini, Bovine; Mus musculus, Mouse; Pan troglodytes, Chimpanzee; Rattus norvegicus, Rat. **F–H** RT–qPCR analysis of circMAP3K4 expression after amplification with oligo (dT)18 primers, RNase R treatment and actinomycin D treatment, respectively. **I** CircMAP3K4 distribution detected by cytoplasmic and nuclear RNA fractionation. GAPDH and U3 were employed as positive controls in the cytoplasm and nucleus, respectively. **J** Fluorescence in situ hybridization showed the predominant cytoplasmic distribution of circMAP3K4 (Scale bar, 20 μm). All experiments were repeated at least three times. Data are shown as mean ± standard deviation (SD), * *p* < 0.05, ** *p* < 0.01, and *** *p* < 0.001 in two-way ANOVA (E) and t-test (C, D). cMAP3K4, circMAP3K4; mMAP3K4, MAP3K4 mRNA

The translation of non-coding RNAs remains elusive, and circRNAs have distinct open reading frame (ORF) patterns. To investigate highly expressed circRNAs with coding potential in HCC, we analyzed the CIRCpedia database which includes millions of circRNA data from 16 paired HCC and non-neoplastic liver tissues [25]. Overall, 116 circRNA candidates were identified with the following inclusion criteria: (1) species conservation, (2) length < 2000nt suitable for further investigations, (3) relatively high levels (FPM > 0.1) in HCC but low levels (FPM < 0.1) in liver tissues, (4) availability of a good detection tool such as MapSplice, and (5) good reproducibility, observed in more than 3 HCC samples (Fig. 1A, left panel). Among these circRNAs, circMAP3K4, circNUFIP2, circARAP2, and two circSLC8A1s also had a potential ORF, m6A modifications, and IRESs, according to the CircBank database (Figure S1A, S1B) [26]. After validating in the CircRNAdb database and SYSUCC data, circMAP3K4 was chosen for further study as a highly expressed circRNA in HCC with translational potential (Fig. 1A, right panel; Figure S1C) [27]. Of note, circMAP3K4 was detected at significantly higher levels in HCC according to the CIRCpedia database (Fig. 1B). All 6 HCC samples showed the increased circMAP3K4 expression compared with the corresponding liver tissues (Fig. 1C). But further detection of MAP3K4 mRNA revealed that there is no significant difference between HCC and the adjacent liver tissues (Figure S1D).

### CircMAP3K4 characteristics in HCC

According to circBase, circMAP3K4 (*hsa_circ_0078619, chr6:161,469,647–161,471,011)* is derived from exon 3 of the *MAP3K4* gene (Fig. 1D, upper panel) [28]. To confirm that circMAP3K4 was circular, we carried out sanger sequencing to verify the specific back-splicing junction sequence (Fig. 1D, lower panel). Additionally, we also found a SNP (Figure S2A). The exon 3 sequences from which circMAP3K4 derived are conserved in various species (Fig. 1E). Compared to random hexamer primers, oligo (dT)_18_ primers could not efficiently amplify circMAP3K4, but the MAP3K4 mRNA (mMAP3K4) was not affected (Fig. 1F). In addition, circMAP3K4 was more resistant to RNase R than mMAP3K4, indicating that circMAP3K4 is not linear (Fig. 1G). CircMAP3K4 was also more stable than mMAP3K4 after actinomycin D treatment (Fig. 1H). Northern blot assay further confirmed circMAP3K4 by specific junction probe (Figure S2B). We next performed cell component extraction and FISH assays, which showed that circMAP3K4 was predominantly located in the cytosol (Fig. 1I, J). Collectively, these results revealed that circMAP3K4 is a stable and circular transcript in HCC cells.

### Clinical implications of circMAP3K4 expression in HCC

**Fig. 2:**
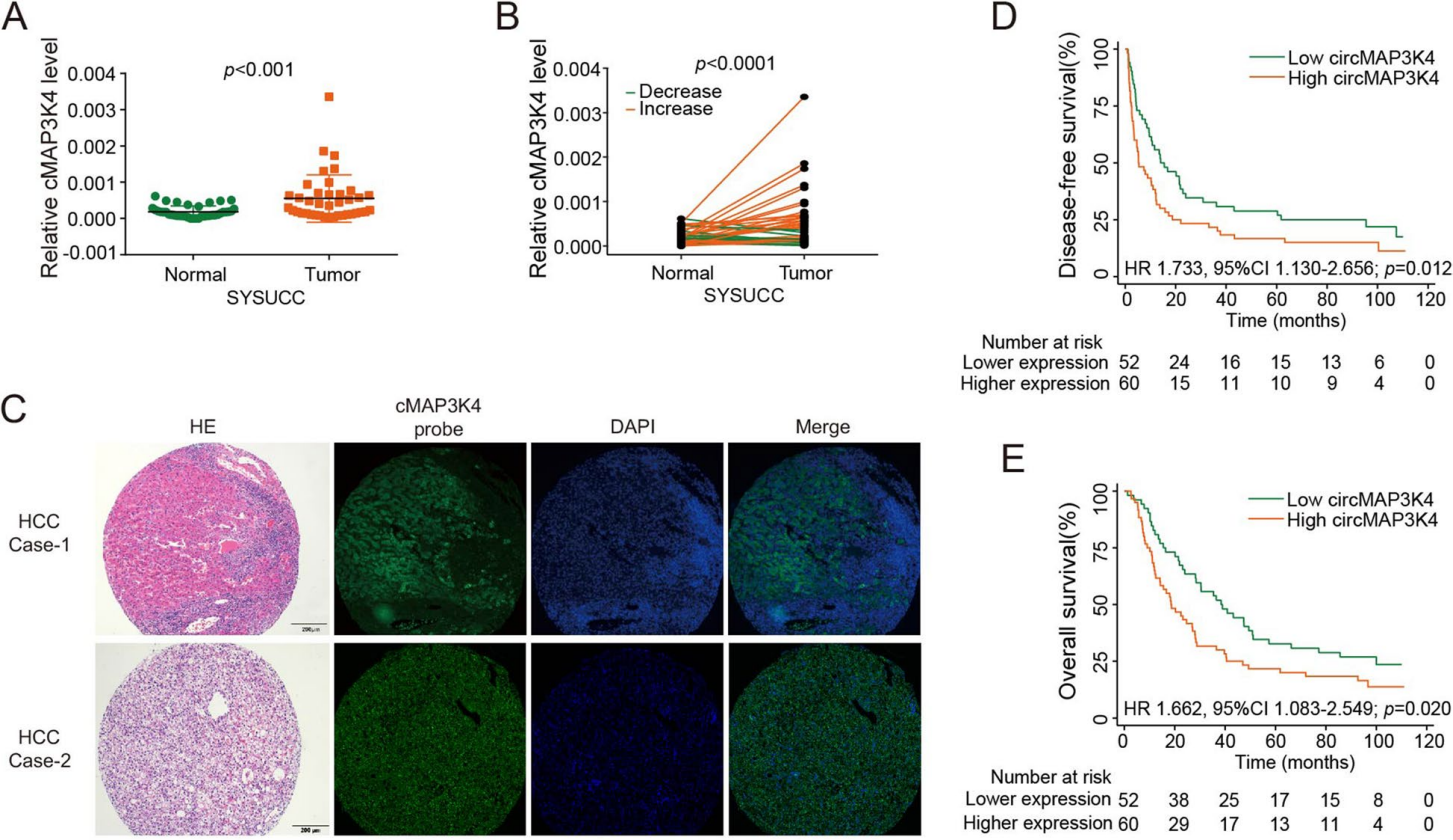
Clinical implications of circMAP3K4 expression in HCC. **A** CircMAP3K4 expression in HCC (*n* = 39) and liver tissues (*n* = 39) from Sun Yat-sen University Cancer Center (SYSUCC). **B** CircMAP3K4 expression in paired HCC and adjacent liver tissues from SYSUCC (*n* = 39). **C** Representative H&E and fluorescence in situ hybridization images of circMAP3K4 RNA in HCC at 20 × . **D**, **E** The disease-free survival and overall survival of HCC patients with high versus low circMAP3K4 levels in SYSUCC samples. cMAP3K4, circMAP3K4; SYSUCC, Sun Yat-sen University Cancer Center; H&E, hematoxylin–eosin staining; HCC, hepatocellular carcinoma; HR, hazard ratio; CI, confidence interval

Consistent with the databases, we further validated that circMAP3K4 was elevated in tumor tissues using 39 paired HCC tissues from Sun Yat-sen University Cancer Center (Fig. 2A, B). Both public data and our cohort suggest circMAP3K4 is upregulated in HCC tissues. Moreover, Hematoxylin and Eosin (H&E) staining and fluorescence in situ hybridization (FISH) assay confirmed the circMAP3K4 expression in HCC (Fig. 2C).

To better understand the role of circMAP3K4 in HCC, we determined circMAP3K4 levels in a cohort of 112 HCC cases from our institute. The circMAP3K4 high- and low-expression cut-offs were determined by receiver operating characteristic (ROC) curve analysis. As shown in Table S1, similar clinical characteristic distributions were observed in the high and low circMAP3K4 subgroups (*p* > 0.05, Chi-square test), except aspartate aminotransferase (AST) (*p* = 0.009). Next, Kaplan–Meier analysis revealed that HCC patients with low circMAP3K4 had superior disease-free survival (DFS, HR = 1.733, 95% CI 1.130–2.656, *p* = 0.012, Fig. 2D) and overall survival (OS, HR = 1.662, 95% CI 1.083–2.549, *p* = 0.020, Fig. 2E) versus those with high circMAP3K4 expression. Furthermore, multivariate analysis using the Cox proportional hazards model demonstrated that circMAP3K4 expression was an independent adverse prognostic factor for HCC patients (Table S2).

### CircMAP3K4 encodes the peptide circMAP3K4-455aa

**Fig. 3:**
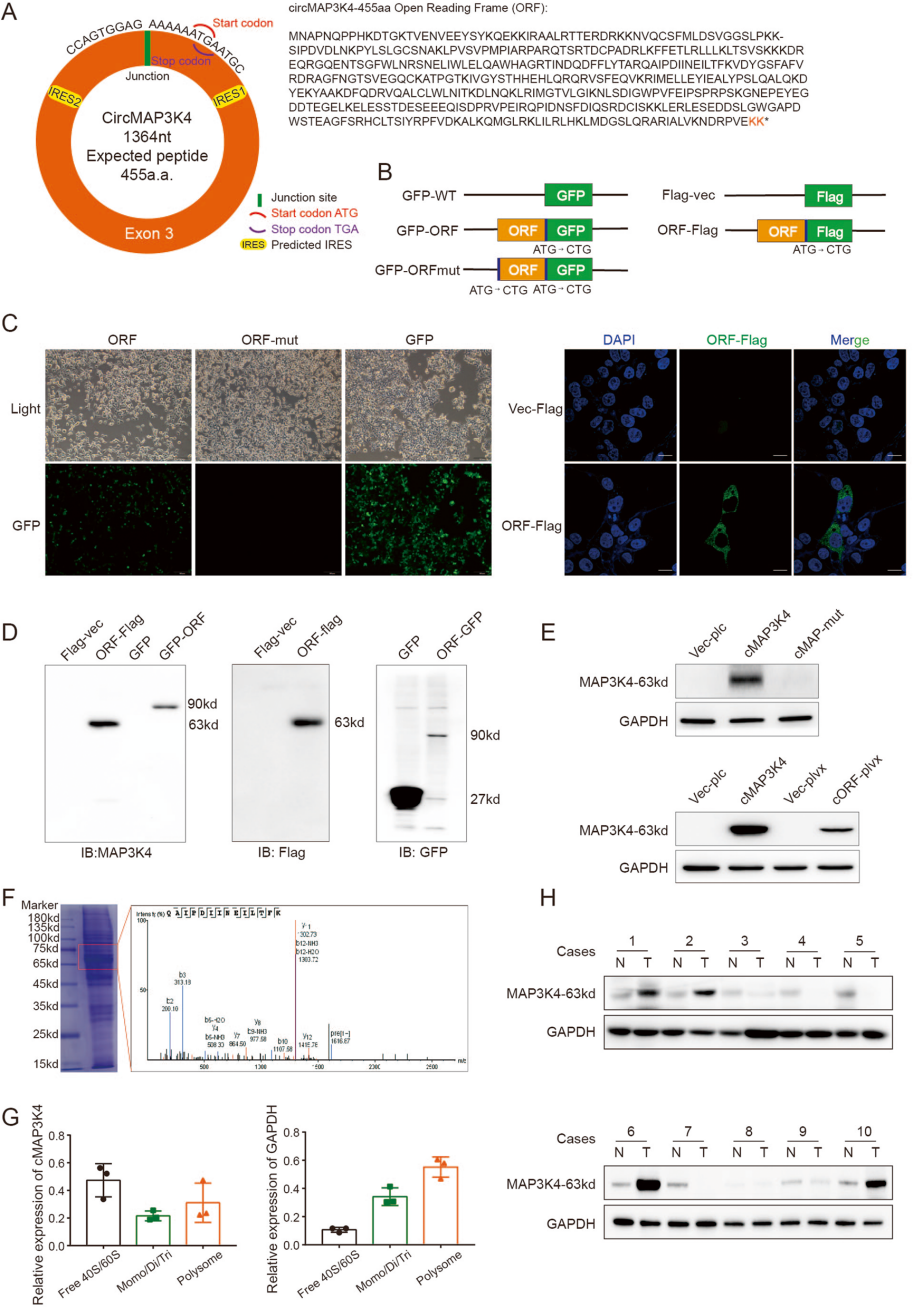
CircMAP3K4-455aa is a novel peptide encoded by circMAP3K4. **A** Schematic illustrations of circMAP3K4 (left panel) with ORF amino acid sequence (right panel). **B** Schematic illustrations of plasmid constructs for circMAP3K4-ORF fused with GFP or Flag tag. **C** Immunofluorescence staining showing HEK293T cells transfected with circMAP3K4-ORF or circMAP3K4-ORF-mut. **D** Western blot analysis of circMAP3K4-455aa expression using anti-MAP3K4, anti-flag, or anti-GFP antibodies. **E** Western blot analysis of circMAP3K4-455aa expression in circMAP3K4 and circMAP3K4-ORF overexpressed cells. **F** LC–MS/MS analysis of the MAP3K4 peptide sequence. **G** Sucrose fractionation assays examining circMAP3K4 distribution with ribosomes with GAPDH as internal control. **H** Western blot analysis of circMAP3K4-455aa expression in 10 cases of HCC and paired liver tissues. ORF, open reading frame; IRES, internal ribosome entry site; GFP, green fluorescent protein; cMAP3K4, circMAP3K4; cMAP-mut, circMAP3K4-mut; cORF, circMAP3K4-ORF; LC–MS/MS, liquid chromatography with tandem mass spectrometry

CircRNAdb database analyses suggested a strong possibility that circMAP3K4 encoded a novel peptide, and the putative ORF (designated as circMAP3K4-ORF) was identified (Fig. 3A). To explore whether the putative circMAP3K4-ORF is active, we constructed a series of plasmids for further function studies (Fig. 3B). CircMAP3K4-ORF was fused to the N terminus of GFP containing a mutated start codon (GFPmut). We observed GFP expression in ORF-GFPmut transfected cells, but not when the ORF had a start codon mutation (ATG to CTG, ORF-mut) (Fig. 3C, left panel). We also confirmed the circMAP3K4-ORF fused with a flag-tag by immunofluorescence (IF) (Fig. 3C, right panel). Additionally, western blot (WB) analysis using anti-GFP and anti-flag antibodies showed that circMAP3K4 could encode a specific peptide weighted around 63kd in the circMAP3K4-ORF groups, but not in the ORF-mut group (Fig. 3D).

To validate the molecular weight of the peptide (circMAP3K4-455aa), we engineered the circMAP3K4-ORF into a prokaryotic system that lacked complex posttranslational modifications. Our results showed that the weight of the peptide mildly increased, possibly due to structural alterations (Figure S2C).

Because the peptide encoded by circMAP3K4 has only two unique amino acids at its C terminal, it is too short to produce a specific antibody to detect this unique peptide. We identified an antibody (ab40784, Abcam) that recognized the 150-250aa of MAP3K4, which, coincidentally, is totally contained in the circMAP3K4-455aa peptide (Figure S2D). To test whether this antibody could also specifically recognize circMAP3K4-455aa, we incubated cell lysates transfected with circMAP3K4 and circMAP3K4-ORF-flag with the anti-MAP3K4 antibody. Our data showed that the antibody could detect a specific band that was the same as the one detected by anti-flag (Fig. 3D, E), but was absent in the circMAP3K4-mut group. Additionally, circMAP3K4-455aa expression markedly decreased when it was co-transfected with circMAP3K4 siRNAs (Figure S2E). To exclude the possibility that this peptide is from the linear MAP3K4 mRNA, Mass spectrometry (MS) analysis of the SDS-PAGE gel around 65kd further confirmed that the circMAP3K4-455aa peptide was expressed from its circular type (Fig. 3F). Sucrose gradient fractionation analysis showed that a proportion of circMAP3K4 sedimented with the polysome fractions, indicating circMAP3K4 was active in translational process (Fig. 3G, S2F). In our WB results, 7/20 HCCs showed higher expression of circMAP3K4-455aa than those in the paired adjacent liver tissues (Fig. 3H, S2G). Taken together, these results revealed that circMAP3K4 encoded the novel peptide circMAP3K4-455aa.

### m6A modification promotes circMAP3K4 translation

**Fig. 4:**
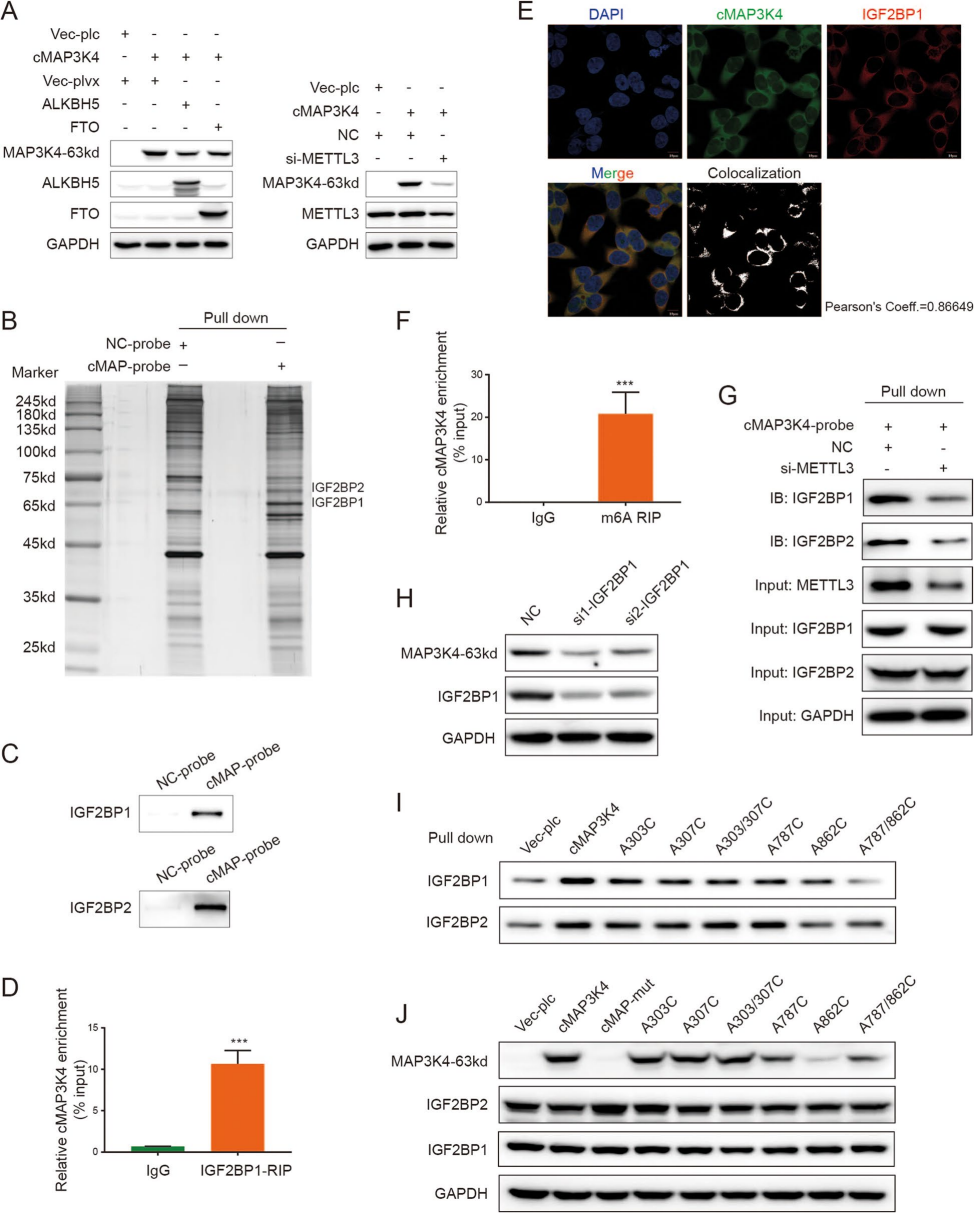
IGF2BP1 mediated circMAP3K4 translation by recognizing m6A modifications. **A** Western blot analysis for circMAP3K4-455aa expression after overexpressing ALKBH5 or FTO (left panel) or knocking down METTL3 (right panel). **B** Silver staining of circMAP3K4 probe pull down products. **C** LC–MS/MS and immunoblot assay for circMAP3K4 interaction with IGF2BP1 or IGF2BP2, respectively. **D**, **E** IGF2BP1 RIP (**D**) and IF-FISH (**E**) verified the IGF2BP1 interaction with circMAP3K4. **F** MeRIP revealed abundant m6A modification of circMAP3K4, normalized to IgG. **G** RNA pull down assay verification that the interaction between IGF2BP1 and circMAP3K4 depends on m6A modification. **H** IGF2BP1 knockdown decreased circMAP3K4-455aa expression. **I**, **J** Immunoblot analyses and western blot of complexes pulled down by the circMAP3K4 probe with various m6A site mutants. cMAP3K4 or cMAP, circMAP3K4

Since circRNAs lack a cap structure, they use two cap-independent approaches for translation: IRESs and m6A modification [19]. According to the circRNAdb, there are two potential IRESs (IRES-1: 83–210 and IRES-2: 1166–1302) in circMAP3K4, so we separately cloned these two IRESs into IRES luciferase reporters. We found that neither of them could initiate luciferase expression (Figure S3A). We further tested whether m6A could regulate circMAP3K4 translation. The overall m6A levels in HCC cells were altered by knocking down the m6A writer METTL3 or overexpressing the erasers ALKBH5 and FTO (Figure S3B). Interestingly, circMAP3K4-455aa expression decreased when m6A levels were downregulated (Fig. 4A). Moreover, SRAMP database analysis showed that circMAP3K4 had a high potential for m6A modifications (Figure S3C) [29]. These results indicate that m6A may be involved in circMAP3K4 translation.

Given the above results, we wondered how the m6A modification regulated circMAP3K4 translation. Using an RNA pull down assay and the followed mass spectrometry analysis for the products, we observed that circMAP3K4 interacted with IGF2BP1 and IGF2BP2, which could act as m6A readers (Fig. 4B). Immunoblot and RNA immunoprecipitation (RIP) further confirmed these interactions (Fig. 4C, D, S3D). Furthermore, immunofluorescence and fluorescence in situ hybridization (IF-FISH) assays demonstrated colocalization of endogenous circMAP3K4 and IGF2BP1 or IGF2BP2 in the cytoplasm (Fig. 4E, S3E). Moreover, our meRIP assay confirmed the enrichment of m6A modifications in circMAP3K4 (Fig. 4F). After knocking down METTL3, circMAP3K4 interaction with IGF2BP1 or IGF2BP2 markedly decreased, suggesting that the interactions depended on m6A (Fig. 4G). Further investigation revealed that IGF2BP1, but not IGF2BP2, could regulate circMAP3K4-455aa expression without affecting circMAP3K4 RNA levels (Fig. 4H, S3F).

As indicated by the SRAMP database, there were multiple sites with a high possibility of m6A modification in circMAP3K4 (Figure S3C). Subsequently, we investigated which circMAP3K4 sites were involved in translation. We selected the top four high confidence m6A sites and mutated the A (adenine) into C (cytosine). We found that the A862C and A787/862C mutation significantly downregulated circMAP3K4-455aa expression, while the other mutations had only minor effects (Fig. 4I, S3G). Consistent with these results, m6A mutations at A862C and A787/862C significantly reduced the interaction between IGF2BP1 and circMAP3K4 (Fig. 4J). Collectively, these data indicated that circMAP3K4 translation is mediated by IGF2BP1 through m6A modification.

### CircMAP3K4-455aa promotes HCC growth *in vitro* and *in vivo*

**Fig. 5:**
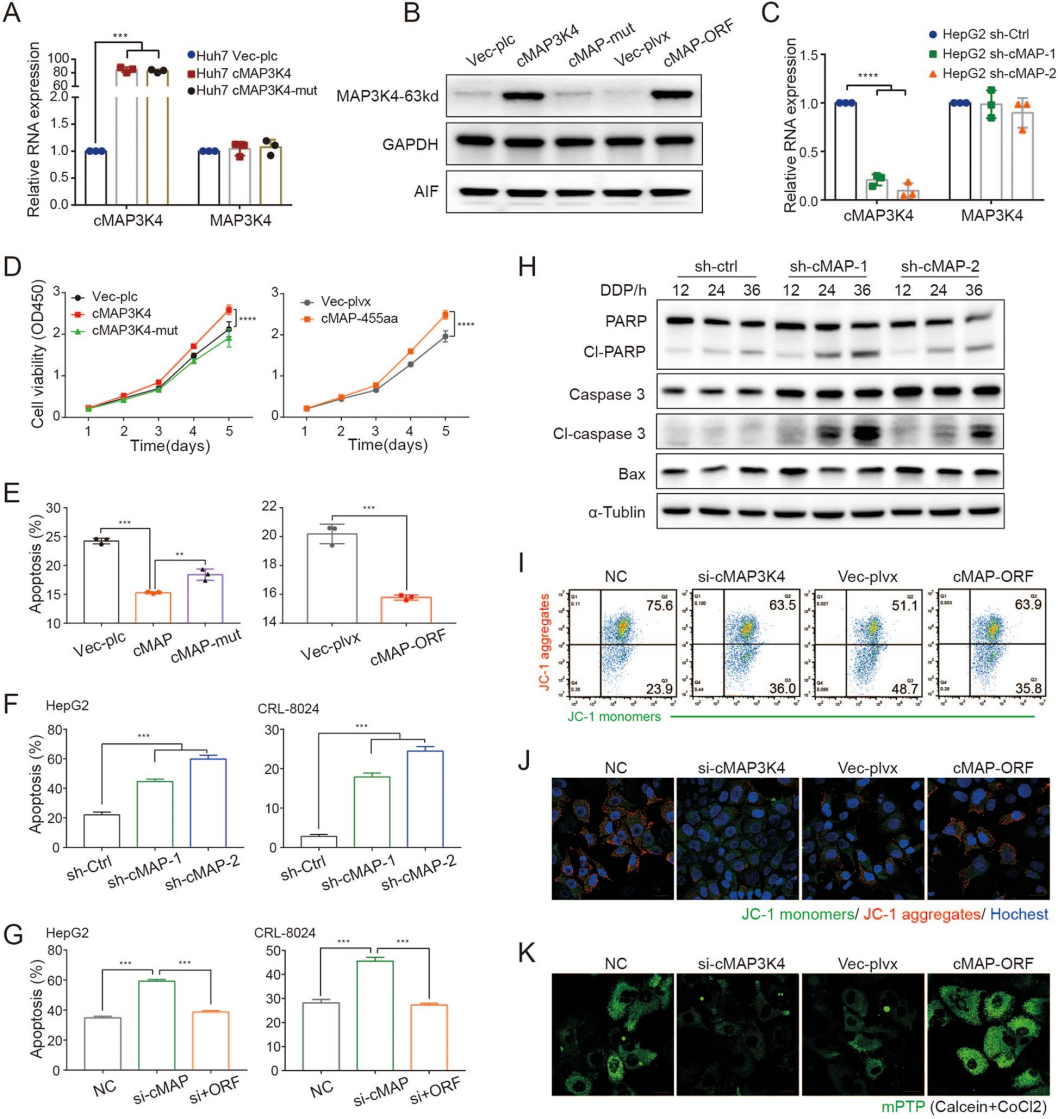
CircMAP3K4-455aa prevents HCC cell apoptosis. **A**-**C** RT-qPCR and western blot analyses of circMAP3K4 or circMAP3K4-455aa in the indicated cells. **D** CCK8 assays for HCC cell proliferation after circMAP3K4 or circMAP3K4-455aa overexpression with indicated controls. **E**, **F** Annexin V/PI staining, an apoptosis indicator, in each group. **G** Annexin V/PI cell staining after circMAP3K4-455aa rescue. **H** Western blot analyses of apoptosis-associated proteins after circMAP3K4 knockdown. **I**, **J** Representative flow cytometry (**I**) and confocal images (**J**) stained with JC-1. **K** Representative confocal images with mPTP. cMAP3K4 or cMAP, circMAP3K4; sh-cMAP-1/2, sh-circMAP3K4-1/2; CCK8, cell counting kit-8; JC-1, 1,1’,3,3’‐tetraethylbenzimi‐dazoylcarbocyanine iodide; mPTP, mitochondrial permeability transition pore. All experiments were repeated three times, data are shown as mean ± SD, * *p* < 0.05, ** *p* < 0.01, and *** *p* < 0.001 in t-test (**A**, **C**, **E**–**G**) or two-way ANOVA (**D**)

Given that high circMAP3K4 expression was linked to HCC patients’ prognosis, we further investigated the biological functions of circMAP3K4 and circMAP3K4-455aa in HCC with a series of *in vitro* and *in vivo* experiments. CircMAP3K4 could function as a non-coding circRNA or a coding peptide. To determine which form is essential for HCC cell growth, we attempted to construct stable HCC cell lines overexpressing circMAP3K4, circMAP3k4-mut, or circMAP3K4-ORF (Fig. 5A, B). To further assess the effects of circMAP3K4 and circMAP3K4-455aa in HCC, we validated two short hairpin RNAs (shRNAs), sh-circMAP3K4-1 and sh-circMAP3K4-2, which specifically targeted circMAP3K4 but did not disturb the host MAP3K4 (Fig. 5C, S4A). We found that circMAP3K4 knockdown remarkably decreased HCC cell growth, while circMAP3K4 or circMAP3K4-455aa overexpression promoted their growth (Fig. 5D, S4B). As the growth of cells is a balance of proliferation and death, we first analyzed the cell cycle in circMAP3K4-455aa overexpressing groups to understand the basis of circMAP3K4-enhanced growth. We observed no significant differences between the circMAP3K4-455aa and control groups in phase distribution (Figure S4C). Systemic chemotherapy such as cisplatin is one of the few alternatives for HCC management, unfortunately, it provides only marginal benefits due to the extreme chemo-resistance [30]. Thus, we further examined cisplatin-induced apoptosis. We found that knocking down circMAP3K4 increased apoptosis in HCC cells, while overexpressing circMAP3K4 decreased the effect, except in circMAP3K4-mut groups (Fig. 5E, F). Moreover, after downregulating circMAP3K4, supplementing circMAP3K4-455aa rescued cell viability in this apoptosis assay (Fig. 5G). WB analysis also indicated more apoptosis in the circMAP3K4 knockdown group (Fig. 5H), and TUNEL and Hoechst 33342 staining showed more cell death after silencing circMAP3K4 (Figure S4D). Moreover, circMAP3K4 could prevent HCC cells from death with oxaliplatin treatment (Figure S4E). During apoptosis, the mitochondrial permeability transition pore (mPTP) opens and decreases mitochondrial membrane potential. Both the JC-1 assay for mitochondrial membrane potential and the mPTP assay supported a protective role of circMAP3K4-455aa in HCC cell apoptosis (Fig. 5I-K, S4F). Collectively, our findings provide evidence that HCC cell resistance to cisplatin-induced apoptosis is at least partly attributable to the circMAP3K4-coding peptide.

**Fig. 6:**
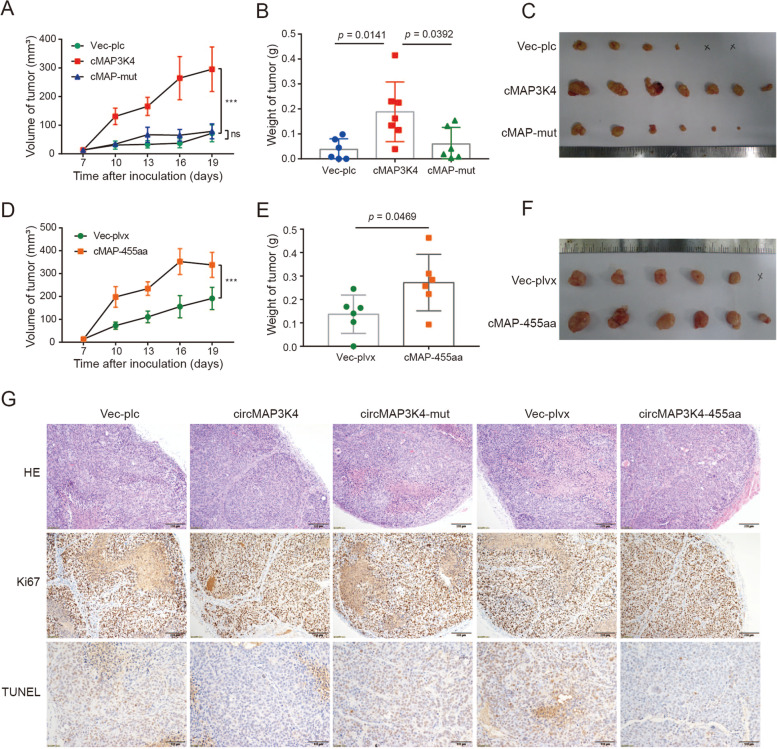
CircMAP3K4-455aa promotes HCC growth in vivo. **A**-**C** Huh7 xenograft growth (**A**), tumor weights (**B**), and photographs (**C**) of the circMAP3K4 (*n* = 7), circMAP3K4-mut (*n* = 6), and control groups (*n* = 6). **D**-**F** Huh7 xenograft growth (**D**), tumor weights (**E**), and photographs (**F**) of the circMAP3K4-455aa (*n* = 6) and vector groups (*n* = 6). **G**. Representative images of HCC tissue stained with HE, Ki67, and TUNEL under the indicated conditions. cMAP3K4 or cMAP, circMAP3K4; HE, Hematoxylin–Eosin staining; TUNEL, TdT-mediated dUTP nick end labeling. * *p* < 0.05, ** *p* < 0.01, and *** *p* < 0.001 in t-test (**B**, **E**) or two-way ANOVA (**A**, **D**)

In line with our in vitro data, our in vivo inoculation data also showed that tumor growth was significantly enhanced after circMAP3K4 or circMAP3K4-ORF overexpression, but not after circMAP3K4-mut overexpression, indicating that the coding circMAP3K4 promoted biological function in HCC (Fig. 6A-F). In comparison with the circMAP3K4-mut group, less necrosis was observed in the circMAP3K4 and circMAP3K4-455aa groups, as observed through Hematoxylin–Eosin (HE) staining (Fig. 6G). Consistent with the HE staining, we found less TUNEL signals in the circMAP3K4 and circMAP3K4-455aa groups (Fig. 6G). These results imply that circMAP3K4 facilitates HCC growth in vivo through the coding peptide circMAP3K4-455aa rather than the circRNA itself.

### CircMAP3K4-455aa prevented HCC cell apoptosis by inhibiting AIF cleavage and nuclear distribution

**Fig. 7:**
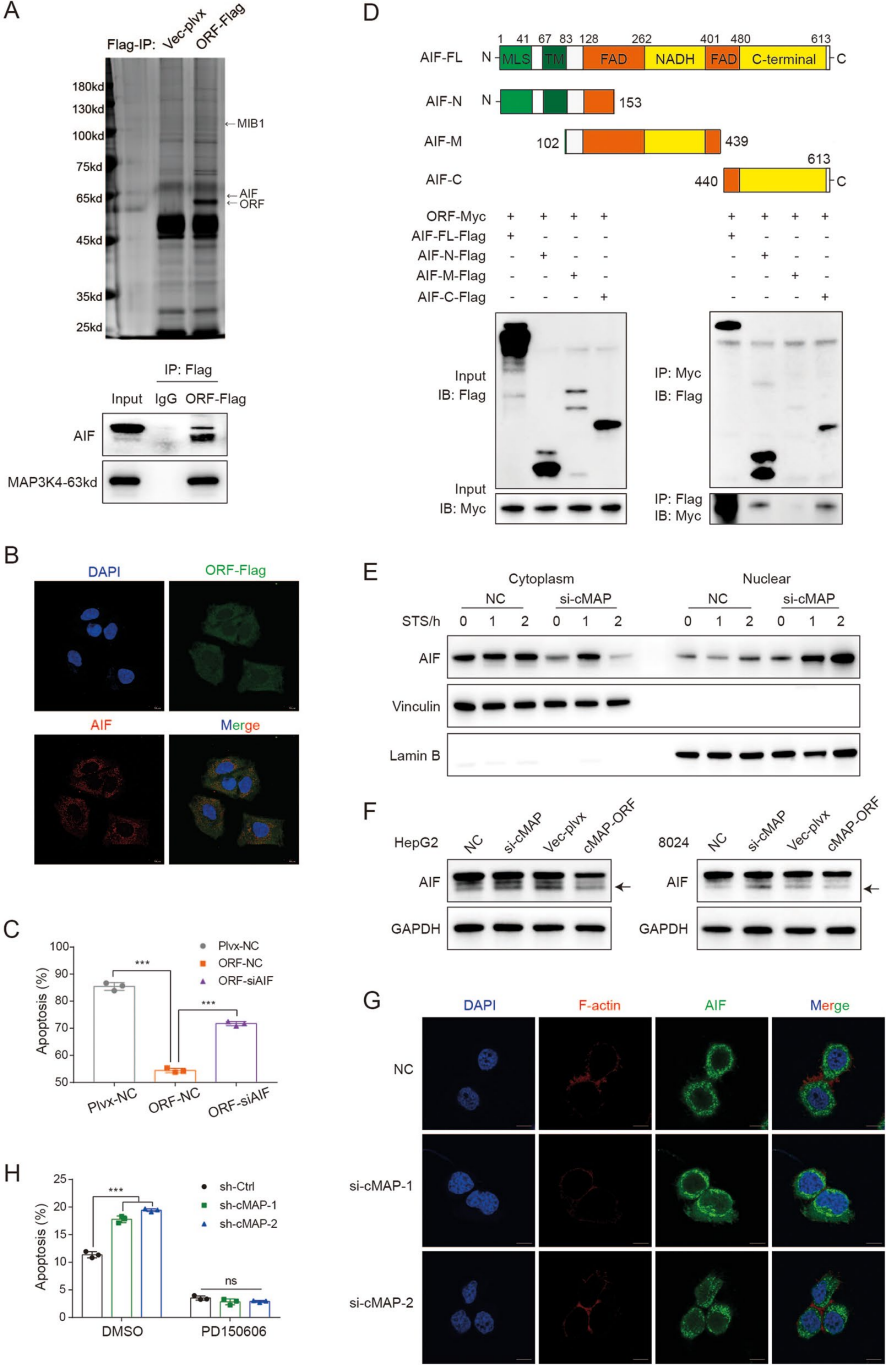
CircMAP3K4-455aa prevented HCC cell apoptosis by inhibiting AIF cleavage and nuclear distribution. **A**, **B** Immunoprecipitation (**A**) and confocal colocalization (**B**) verified AIF interaction with circMAP3K4-455aa. **C** Annexin V/PI staining of rescue experiments after AIF knockdown in HCC cells that overexpress circMAP3K4-455aa. **D** Schematic illustrations of the constructed plasmids (upper panel) and immunoblot analysis (lower panel) of immunoprecipitants after co-transfection with AIF mutant truncations and circMAP3K4-455aa. **E** Representative western blot for AIF in cytoplasmic and nuclear fractions after treatment with STS. **F** Representative western blot for AIF and fragmented AIF (arrow) after exposure to CDDP. **G** Representative confocal images of AIF distribution after STS treatment. **H** Annexin V/PI staining of HCC cells with or without PD150606 treatment. cMAP, circMAP3K4; STS, Staurosporine; CDDP, cisplatin; *** *p* < 0.001 in t-test (**C**, **H**)

To determine the potential molecular mechanism of circMAP3K4-455aa in preventing HCC cells from apoptosis, we used LC–MS/MS to identify proteins that interacted with circMAP3K4-455aa and found that AIF was a potential target associated with cell death (Fig. 7A). We confirmed the interaction using immunoprecipitation (IP), which pulled down the endogenous AIF in two forms: the AIF-precursor and the mature AIF (55-613aa, 62kd) (Fig. 7A).

After translation in the cytosol, the AIF-precursor (1-613aa, 67kd) is cleaved into mature AIF (55-613aa, 62kd) and translocates from the cytosol into the mitochondria [31, 32]. Induction of apoptosis results in further cleavage of mature AIF into soluble AIF (104-613aa, 57kd), which is released from the mitochondria into the cytosol and the nucleus where it promotes DNA cleavage [33–35].

We further validated the colocalization of AIF and circMAP3K4-455aa in the cytoplasm using IF (Fig. 7B). Because AIF plays an important role in cell death, we wondered if circMAP3K4-455aa prevents HCC cells from cisplatin-induced death by interacting with AIF. Our rescue results showed that knocking down AIF abolished the decreased in HCC cell apoptosis caused by overexpressing circMAP3K4-455aa (Figure S5A, 7C). The expression of AIF did not be affected by the overexpression of circMAP3K4 and circMAP3K4-455aa (Fig. 5B). The interaction with mature AIF in the mitochondria prompted us to further identify the subcellular location of circMAP3K4-455aa. Mitochondrial isolation and subsequent immunoblot assays demonstrated that circMAP3K4-455aa was present in the mitochondria (Figure S5B). Together, these results revealed that circMAP3K4-455aa interacted with AIF in the mitochondria.

To investigate which AIF domains potentially interact with circMAP3K4-455aa, we constructed three AIF mutants with N-terminal (1-153aa), middle (102-439aa), or C-terminal (440-613aa) truncations. Co-IP data showed that both the AIF N-terminus and C-terminus could interact with circMAP3K4-455aa (Fig. 7D). Moreover, the interaction of AIF and truncated circMAP3K4-455aa showed that AIF could interact with all parts of circMAP3K4-455aa (Figure S5C).

AIF N-terminal cleavage by calpain is an important step for releasing AIF into the cytosol during apoptosis, initiating the release of cytochrome c and activating caspases [36]. Thus, we further determined whether circMAP3K4-455aa was involved in this process by protecting the AIF N-terminus from cleavage. Our cell fractionation data revealed that more AIF was located in the nuclei of circMAP3K4 knockdown cells treated with staurosporine (STS, an inducer of AIF cleavage) (Fig. 7E). CircMAP3K4 knockdown HCC cells had more soluble AIF after cisplatin treatment, while overexpressing circMAP3K4-455aa had the opposite effect (Fig. 7F). In addition, we found more nuclear distribution of AIF in the circMAP3K4-knockdown group (Fig. 7G). To further identify whether circMAP3K4-455aa was implicated in AIF N-terminal cleavage, we used a calpain inhibitor (PD150606) for further tests. As expected, we found that PD150606 treatment abolished the increased apoptosis in circMAP3K4-knockdown cells (Fig. 7H), indicating that the translated circMAP3K4-455aa peptide could inhibit AIF N-terminal cleavage and nuclear distribution in order to decrease cisplatin-induced cell death.

### MIB1 promotes circMAP3K4-455aa ubiquitination and degradation

**Fig. 8:**
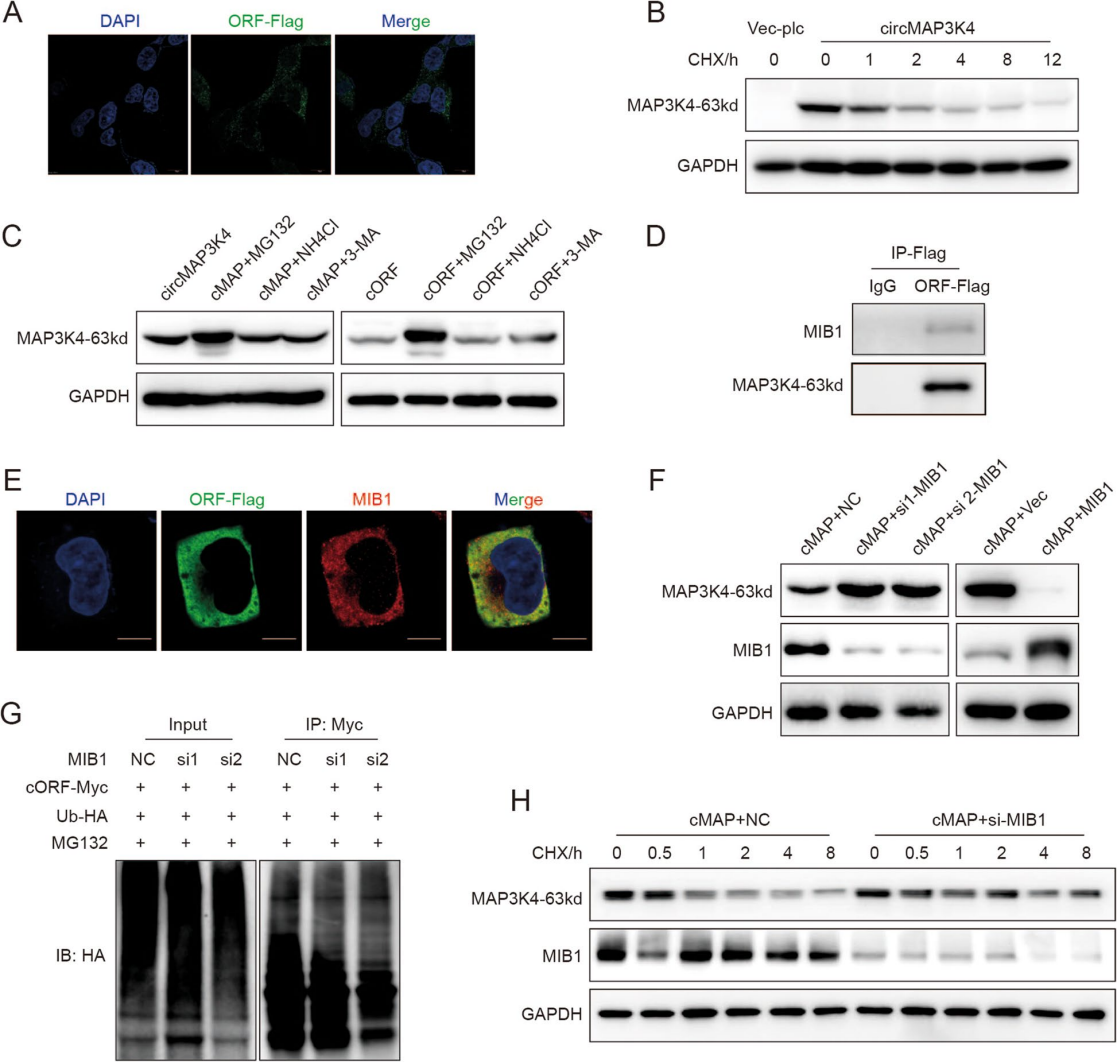
CircMAP3K4-455aa was ubiquitinated by MIB1 and degraded by the ubiquitin–proteasome pathway. **A** Representative confocal images showing the cytoplasmic location of circMAP3K4-455aa. **B** The half-life of circMAP3K4-455aa protein after treatment with cycloheximide. **C** Western blot assay examining circMAP3K4-455aa expression levels after treatment with MG132, NH_4_Cl, or 3-Methyladenine. D, E. Co-IP (**D**) and confocal colocalization (**E**) verified circMAP3K4-455aa interaction with MIB1. **F** Western blot detection of circMAP3K4-455aa after MIB1 knockdown (left panel) and MIB overexpression (right panel). **G** *In vivo* ubiquitylation assay assessing circMAP3K4-455aa ubiquitination after MIB1 knockdown. **H** Detection of the circMAP3K4 half-life after decreasing MIB1 expression. CHX, Cycloheximide; cMAP, circMAP3K4; 3-MA, 3-Methyladenine; IP, Immunoprecipitation

To gain more insights into circMAP3K4-455aa modulation, we further explored circMAP3K4-455aa characteristics. IF staining revealed circMAP3K4-455aa distribution in the cytoplasm (Fig. 8A). CircMAP3K4-455aa dramatically decreased in cycloheximide (CHX)-treated cells, indicating that the peptide was unstable, with a half-life of around 1–2 h (Fig. 8B). To further investigate its degradation, we treated circMAP3K4- or circMAP3K4-455aa-overexpressing cells with MG132 (an inhibitor for ubiquitin–proteasome degradation), NH_4_Cl (an inhibitor for lysosomal degradation), or 3-Methyladenine (3-MA, an inhibitor for autophagy). Our results showed that only MG132 treatment increased circMAP3K4-455aa levels, indicating that the circMAP3K4-455aa peptide was degraded through the ubiquitin–proteasome pathway (Fig. 8C). In addition, the ubiquitylation assay confirmed circMAP3K4-455aa ubiquitin modification (Figure S5D).

Among the interacting proteins identified by LC–MS/MS, circMAP3K4-455aa was highly likely to interact with Mindbomb E3 Ubiquitin Protein Ligase 1 (MIB1). We confirmed their interaction and colocalization in the cytoplasm using co-IP and IF staining (Fig. 8D, E). MIB1 is an E3 ligase and circMAP3K4-455aa is degraded through the ubiquitin–proteasome pathway; thus, we investigated whether MIB1 regulated circMAP3K4-455aa by ubiquitin modification. We found that overexpressing MIB1 decreased circMAP3K4-455aa expression and that silencing MIB1 elevated circMAP3K4-455aa levels (Fig. 8F). After knocking down MIB1, an in vivo ubiquitination assay revealed decreased circMAP3K4-455aa poly-ubiquitin modification with HA-ubiquitin (Fig. 8G). Moreover, the circMAP3K4-455aa half-life was prolonged after MIB1 knock down (Fig. 8H, S5E). These data suggest that MIB1 is essential for the ubiquitination and proteasomal degradation of circMAP3K4-455aa.

## Discussion

Emerging studies have revealed that dysregulated circRNA expression has important roles in the tumorigenesis and progression of human cancers [37–39]. Globally, circRNAs are generally downregulated in a diverse range of tumors. However, a small group of upregulated circRNAs were identified in previous reports [40], suggesting that circRNAs have tissue- or developmental stage-specific expression patterns [41–43]. To explore the function of potential coding circRNAs involved in HCC carcinogenesis and progression, we sought here to characterize highly expressed, translated circRNAs in HCC. Based on strict inclusion criteria, we identified and selected circMAP3K4 for further investigation. CircMAP3K4 was significantly upregulated in HCC and associated with both adverse overall survival and disease-free survival in patients. Importantly, we also found that circMAP3K4 could encode for a protein, circMAP3K4-455aa, and protect HCC cells from cisplatin-induced apoptosis. Our findings underscore a potentially oncogenic role of circMAP3K4 in the development and progression of HCC, although more work is needed to definitively identify more oncogenic or suppressor circRNAs in tumors.

We observed that circMAP3K4 translation was dependent on m6A modification and mediated by IGF2BP1 recruitment. Many endogenous circRNAs have been shown to modulate transcription by sponging microRNAs or interacting with other molecules [44]. Before evidence of protein coding was discovered, circRNAs were generally considered to be non-coding RNAs [45]. Studies regarding the translational relevance of circRNAs, as well as the mechanisms involved in their coding, have been limited [45]. Recent studies have demonstrated that a small set of circRNAs have a natural IRES for encoding peptides, while a few are engaged in m6A-dependent translation [8]. In the current study, we established that circMAP3K4 could be translated into the peptide circMAP3K4-455aa through IGF2BP1 mediated m6A recognition. m6A modification has been shown to promote circRNA translation by recruiting the m6A reader YTHDF3 [24]. Our results further support and broaden m6A-based circRNA translation mechanisms. Numerous studies reported that IGF2BP1 could involve in translation, including virus translation, through stabilizing mRNA, interacting with initiation factor or binding 5'-UTR IRES [46–50]. Notably, Chatterji et al. revealed that IGF2BP1 could affect the translational efficiency of various genes [51]. However, the specific mechanism of IGF2BP1 in circRNAs translation remains elusive and needs further investigation. Taken together, these data suggest that IGF2BP1 could participate in circRNA translation by recognizing m6A modification.

Interestingly, our co-IP and LC/MS–MS data also demonstrated that circMAP3K4-455aa could bind the N-terminus and C-terminus of endogenous AIF. AIF is located in the mitochondria and released after apoptotic stimuli, upon which it translocates to the nucleus and subsequently results in cell death in a caspase-independent manner [52]. Because AIF plays a key role in regulating cell survival or death [36] and HCCs are resistant to conventional chemotherapeutic agents (including cisplatin) [7], we wondered whether circMAP3K4-455aa could prevent HCC cells from cisplatin-induced death in an AIF mediated fashion. Our results revealed that circMAP3K4-455aa interacted with AIF in the mitochondria and decreased AIF nuclear distribution, leading to increased HCC cell survival after exposure to cisplatin. We then investigated which domains of AIF interacted with circMAP3K4-455aa and were involved in cell survival. We found evidence that both the N-terminus and C-terminus could interact with circMAP3K4-455aa in HCC cells. AIF N-terminal cleavage by the calcium-activated protease calpain mediates its release from mitochondria during cell death [53]. Thus, we speculated that circMAP3K4-455aa may be involved in this process by protecting the AIF N-terminus from cleavage, thereby preventing HCC cells from cisplatin-induced death.

## Conclusion

We have provided comprehensive evidence that circMAP3K4 plays a crucial role in HCC cell survival after exposure to cisplatin and is an independent prognostic predictor for HCC patients. CircMAP3K4 can be translated into a peptide via IGF2BP1 recognition-mediated m6A modification. The peptide prevents cisplatin-induced cell death by binding to the AIF N-terminus, decreasing AIF cleavage and nuclear distribution. These findings highlight the prognostic and/or therapeutic implications of the circMAP3K4 encoding peptide in HCC patients.

## Supplementary Information

**Additional file 1.**

## Data Availability

The datasets sharing is available from the corresponding author Mu-Yan Cai on reasonable request.

## Abbreviations

circRNAs: Circular RNAs
HCC: Hepatocellular carcinoma
m6A: N6-methyladenosine
TKIs: Tyrosine kinase inhibitors
miRNA: MicroRNA
IRESs: Internal ribosome entry sites
AIF: Apoptosis inducing factor mitochondria associated 1
ORF: Open reading frame
FISH: Fluorescence in situ hybridization
ROC: Receiver operating characteristic
AST: Aspartate aminotransferase
DFS: Disease-free survival
OS: Overall survival
IF: Immunofluorescence
WB: Western blotting
RIP: RNA immunoprecipitation
IF-FISH: Immunofluorescence and fluorescence in situ hybridization
mPTP: Mitochondrial permeability transition pore
IP: Immunoprecipitation
STS: Staurosporine
CHX: Cycloheximide
MIB1: Mindbomb E3 Ubiquitin Protein Ligase 1
