# Supplementary material for: A novel peptide encoded by N6-methyladenosine modified circMAP3K4 prevents apoptosis in hepatocellular carcinoma
Source: Mol Cancer. 2022 Apr 2;21:93. doi: 10.1186/s12943-022-01537-5 (PMC8976336; doi:10.1186/s12943-022-01537-5)
Supplement: Supplementary file 1 — Additional file 1. [file 12943_2022_1537_MOESM1_ESM.doc]

Supplementary Materials for

**A Novel Peptide Encoded by N6-methyladenosine Modified circMAP3K4 Prevents Apoptosis in Hepatocellular Carcinoma**

Jin-Ling Duan^1,2#^, Wei Chen^3#^, Juan-Juan Xie^1#^, Mao-Lei Zhang^4#^, Run-Cong Nie^1,5#^, Hu Liang^1,6^, Jie Mei^1,5^, Kai Han^1,5^, Zhi-Cheng Xiang^1,2^, Feng-Wei Wang^1^, Kai Teng^7^, Ri-Xin Chen^8^, Min-Hua Deng^1,5^, Yi-Xin Yin^1^, Nu Zhang^4*^, Dan Xie^1,2*^ and Mu-Yan Cai^1,2*^

^#^These authors contributed equally to this work.

*Corresponding authors: Mu-Yan Cai, Dan Xie or Nu Zhang; E-mail: [caimy@sysucc.org.cn](mailto:caimy@sysucc.org.cn), [xiedan@sysucc.org.cn](mailto:xiedan@sysucc.org.cn) or [Zhangnu2@mail.sysu.edu.cn](mailto:Zhangnu2@mail.sysu.edu.cn).

**This file includes:**

Supplementary Materials and methods

Figure S1 to S5

Tables S1 to S3

**Supplementary Materials and methods**

**Cell culture**. The HEK293T and CRL-8024 cell lines were originally obtained from the American Type Culture Collection (ATCC). SNU449 and HepG2 cell lines were kindly gifted by professor Xiaofeng Zheng (Sun Yat-sen University Cancer center). LO2 and Huh7 cell lines were provided by professor Yunfei Yuan (Sun Yat-sen University Cancer center). All cell lines were authenticated. The cells were cultured in Dulbecco’s modified Eagle’s medium (DMEM) supplemented with 10% FBS (Gibco, NY, USA) and 1% Penicillin-Streptomycin. Cells were cultured in a 37 °C incubator (Thermo Fisher Scientific, Waltham, MA, USA) with 5% CO_2_ and 95% air. All cell lines tested negative for mycoplasma.

**Patients and samples**. This study has been approved by the Institutional Review Board of Sun Yat-Sen University Cancer Center (SYSUCC, Guangzhou, China), and the study was conducted in accordance with the Declaration of Helsinki. Written informed consent was obtained from all patients before this study. All primary HCC and paired liver tissue samples were obtained from HCC patients during operations between January 2011 and December 2015 at the Sun Yat-Sen University Cancer Center (SYSUCC), Guangzhou, China. Clinicopathological features of the HCC cohort are summarized in **Table S1**. All patients were followed up with using a regular method. Disease-free survival (DFS) was defined as the time from randomization to recurrence of tumor or death, and overall survival (OS) was determined from the date of surgery to the date of death or the date of the last follow-up visit for survivors.

**Plasmid construction.** Specific circMAP3K4 siRNAs were synthesized by RiboBio (Guangzhou, China) and specifically targeted the junction sequence. shRNAs with specific sequences were obtained from GeneCopeia (MD, USA). The circMAP3K4 overexpression plasmid was constructed by Geneseed (Guangzhou, China). The expression plasmids for circMAP3K4-ORF, full-length AIF, and truncated AIF or circMAP3K4-ORF mutations were generated by amplifying the corresponding cDNA with PCR and cloning it into pLVX-puro-flag-myc or pEGFP-N1 vectors using a Mut Express II Fast Mutagenesis Kit V2 (Vazyme, China). MIB plasmids (p3HA-hMIB1 #33317) were purchased from Addgene.

**Antibodies.** The antibodies used in this study are described as follows: anti-MAP3K4 rabbit mAb (Abcam ab40784, WB 1:1000), anti-Flag rabbit mAb (CST #14793, WB 1:1000, co-IP 1:100), anti-Myc-Tag mouse mAb (CST #2276, WB 1:1000, co-IP 1:100), anti-HA-Tag rabbit mAb (CST #3724, WB 1:1000, co-IP 1:100), anti-GAPDH mouse mAb (Proteintech 60004-1-Ig, WB 1:5000), anti-alpha tubulin rabbit pAb (Proteintech 11224-1-AP, WB 1:5000), anti-IGF2BP1 rabbit pAb (Proteintech 22803-1-AP, WB 1:1000, RIP 4μg/test), anti-IGF2BP2 rabbit pAb (Proteintech 11601-1-AP, WB 1:1000, RIP 4μg/test), anti-m6A rabbit pAb (Synaptic Systems, WB 1:1000, meRIP 4 μg/test), anti-MIB1 rabbit pAb (Proteintech 11893-1-AP, WB 1:1000), anti-AIF rabbit pAb (Proteintech 17984-1-AP, WB 1:1000), Phalloidin-iFluor 555 reagent (ab176756), anti-Tom20 rabbit pAb (Proteintech 11802-1-AP, WB 1:1000).

**Actinomycin D and RNase R treatment**. HCC cells in 6-well plates were treated with 5ug/ml Actinomycin D or DMSO for the indicated durations and collected for RT-qPCR analysis. 2ug total RNA was incubated with 2 U/μg of RNase R (Epicentre Technologies, Madison, WI, USA) for 15 min at 37 °C and transcribed into cDNA for RT-qPCR testing. The primers for RT-qPCR used in this article are listed in **Table S3**.

**Nuclear and cytoplasmic extraction**. Cytoplasmic and nuclear fractions were isolated using the PARIS™ Kit (AM1556, Thermo Fisher Scienbtific, Waltham, USA). According to the manufacturer’s instructions, cells were lysed in Cell Fraction Buffer on ice for 10 min and then centrifugated at 500 × g for 3 min in a low temperature centrifuge. The supernatant was the cytoplasmic fraction. The pellets were then lysed, centrifuged, and collected with Nuclear Fraction Buffer. The primers for RT-qPCR used in this article are listed in **Table S3**.

**Immunofluorescence (IF)**. HCC cells or HEK293T cells in confocal dishes (Corning, United States) were fixed with 4% paraformaldehyde for 15 min and then permeabilized with 0.5% TritonX-100 in PBS (PBST) for 15 min. The antigens were further blocked with 5% BSA for 30 min. The cells were next incubated with primary antibody overnight at 4°C. The next day, dishes were washed with PBST three times, incubated with fluorescent-dye conjugated secondary antibody for 1 h at room temperature, and stained with DAPI (Beyotime, Shang-hai, China) before imaging. The images were acquired using a fluorescence microscope (OLYMPUS FV1000 confocal microscopy, Japan) and analyzed with the software provided by the manufacturer.

**RNA fluorescence in situ hybridization (FISH)**. The cells were seeded in confocal dishes (Corning, United States) and fixed with a 3:1 mixture of methanol and acetic acid for 30 min. Then the dishes were sequentially incubated with 70%, 80%, and 100% ethanol for 2 min each. At the same time, the circMAP3K4 probe was denatured at 80 °C for 10 min and then immediately cooled on ice. After mixing with hybridization liquid, 10 μl of probe liquid was dropped onto the dish, which was carefully covered and sealed with sealing film. The dishes were incubated in a moist chamber at 37 °C overnight. The next day, the dishes were washed using 100% ethanol for 2 min and then twice using 2× SSC. The conjugation process followed the Alexa Fluro 488 Tyramide SuperBoost^TM^ Kit instructions (Invitrogen, Carlsbad, CA, USA). DAPI (Beyotime, Shang-hai, China) was used to stain the nuclei. CircMAP3K4 images were obtained using fluorescence microscopy (OLYMPUS FV1000 confocal microscopy, Japan). The circMAP3K4 oligonucleotide-modified probe was synthesized by RuiBiotech (Guangzhou, China). Sequences for probes used in this article are listed in **Table S3**.

**RNA pull down**. HCC and HEK293T cell lysates with overexpression of circMAP3K4 or mutant plasmids were incubated with the denatured biotin-labeled probe at 4°C overnight. The next day, 25 μl of streptavidin magnetic beads (Invitrogen, Carlsbad, CA, USA) were added and rotated for 4–5 h at 4 °C. After the incubation, beads were washed with washing buffer 5 times and denatured with SDS lysate for silver staining, mass spectrometry, or western blot detection.

**RNA-binding protein immunoprecipitation (RIP)**. Immunoprecipitations were performed according to the Magna RIP RNA-Binding Protein Immunoprecipitation Kit instructions (Millipore, MA, USA). IGF2BP1, IGF2BP2, or m6A antibodies were used. The immunoprecipitated RNAs were subjected to RT-qPCR analysis.

**In vivo ubiquitylation assay**. Cells were transiently transfected with plasmids expressing HA-Ub for 24 h, with or without myc-tag vectors. After treatment with MG132 (MCE, Shanghai, China) for 8 h, cells were washed with PBS and then lysed in 100 μl denaturing buffer (50 mM Tris–HCl pH 8.0, 10 mM EDTA, 1% SDS) by boiling for 10 min. The lysates were made up to 1 ml with lysis buffer and ultrasonication, immunoprecipitated using 4 μg anti-Myc tag antibody, and then subjected to WB with anti-HA or anti-ubiquitin antibodies to test for circMAP3K4-455aa ubiquitylation.

**Cycloheximide (CHX) chase assay.** CircMAP3K4-455aa stability was determined using the CHX chase assay. CircMAP3K4-overexpressed cells were seeded in 6-well plates for 24 h, treated with CHX (100 μg/ml) for the indicated time, and then collected and lysed for western blot analysis.

**Coimmunoprecipitation assay.** Cells were transfected with the indicated plasmids for 48 h, then collected and lysed in 1 ml TNE IP lysis buffer (50mM Tris–HCl pH 7.4, 150 mM NaCl, 1 mM EDTA, 0.5% NP40, 10% glycerol). Co-IP assays were performed using anti-Flag tag and IgG antibodies. The immune complexes were captured by Protein A/G Magnetic Beads (#88802, Thermo Fisher Scientific, Waltham, USA). The co-IP products were used for western blotting with the indicated antibodies or staining with silver for the MS assay.

**Apoptosis assay.** HCC cells were transduced with lentivirus to deliver vectors overexpressing genes or encoding shRNAs. After pretreatment with cisplatin (5 μg/ml) for 24 h, cells were collected and stained with Annexin V-APC and propidium iodide. The cells were then tested using flow cytometry and the results were analyzed with CytExpert. Each experiment was conducted at least in triplicate. Statistical differences were analyzed based on the sum of the proportions of cells in early apoptosis and late apoptosis categories.

**In vivo xenograft studies.** Athymic nude mice were purchased from Vital River Laboratories (Beijing, China) and housed under standard SPF conditions at the Center of Experimental Animals of Sun Yat-Sen University Cancer Center. Five million Huh7 cells with circMAP3K4, circMAP3K4-mut, or circMAP3K4-ORF overexpression and control cells were inoculated subcutaneously in the right flank of nude mice. Tumors were measured twice weekly with calipers, and tumor volumes were calculated using the formula 1/2 × (width^2^ × length). Tumors were collected at the indicated time. After photographing and weighing, tumors were fixed and paraffin-embedded for further analysis.

**Statistical analysis**. Statistical analyses were performed using the SPSS 20.0 software (SPSS, Chicago, IL, USA). Receiver operating characteristic (ROC) curve analysis was applied to determine the cut-off score for high circMAP3K4 expression. Associations between circMAP3K4 expression and the clinicopathological parameters were analyzed using the Pearson chi-square test. The relationship between circMAP3K4 expression and patients’ DFS or OS were estimated using the log-rank test. Multivariate survival analyses were performed using the Cox proportional hazards regression model. Differences between the experimental and control groups were tested using a two-tailed Student’s t-test. Data are presented as mean ± SD and a P value of less than 0.05 was considered to be statistically significant. **P* < 0.05, ***P* < 0.01, and ****P* < 0.001 versus the corresponding controls.

**Supplementary Figures**

**Figure S1**


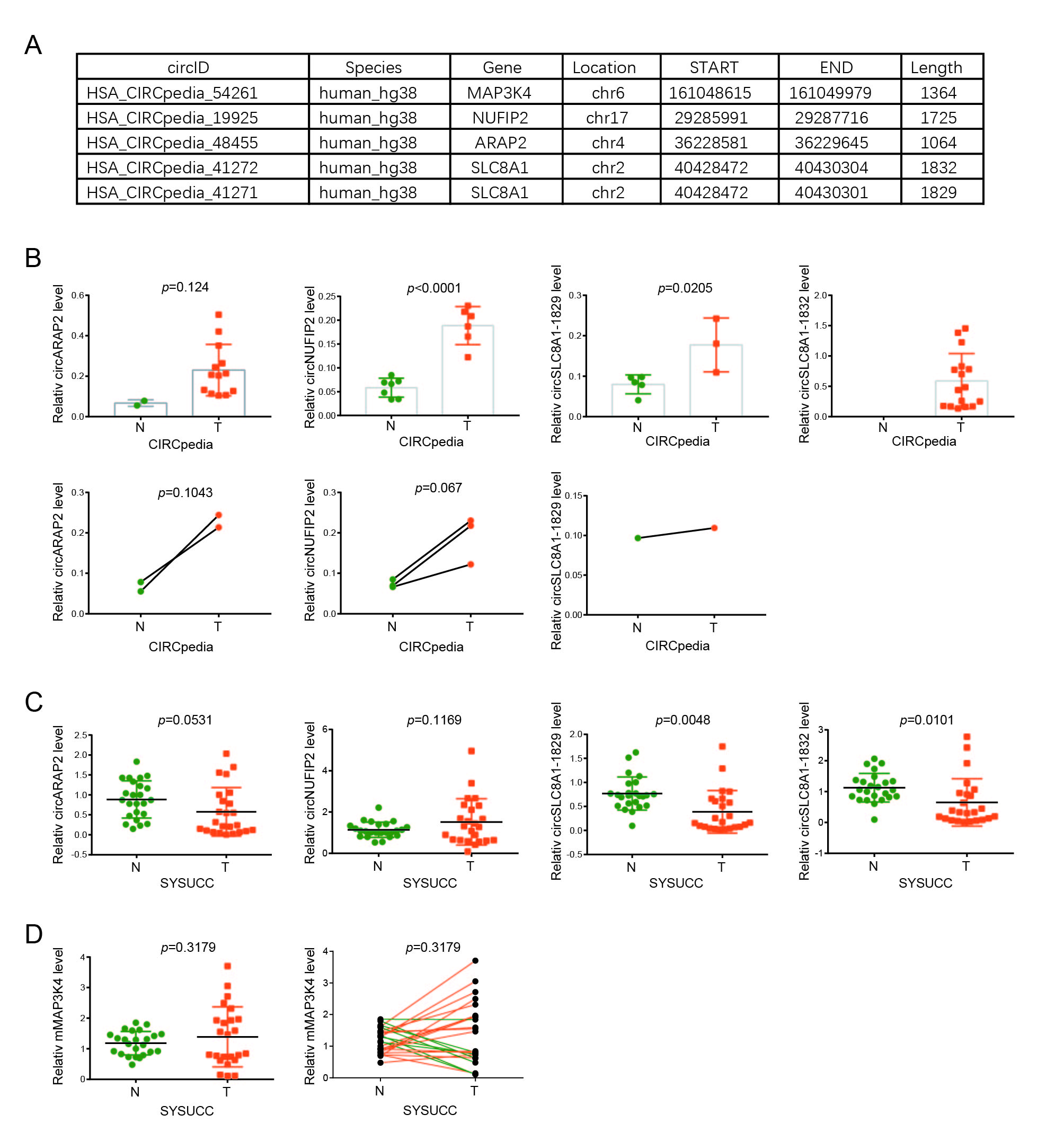


**Figure S1. Selection of** **highly expressed candidate circRNAs in HCC.** A. Information pertaining to the highly expressed candidate circRNAs in HCC. B. The expression of four highly expressed candidate circRNAs in HCC and non-neoplastic liver tissues from the CIRCpedia database. C. The expression of four highly expressed candidate circRNAs in HCC and non-neoplastic liver tissues (n=24) from SYSUCC. D. The expression of MAP3K4 mRNA in paired HCC and non-neoplastic liver tissues (n=24) from SYSUCC. mMAP3K4, MAP3K4 mRNA.

**Figure S2**


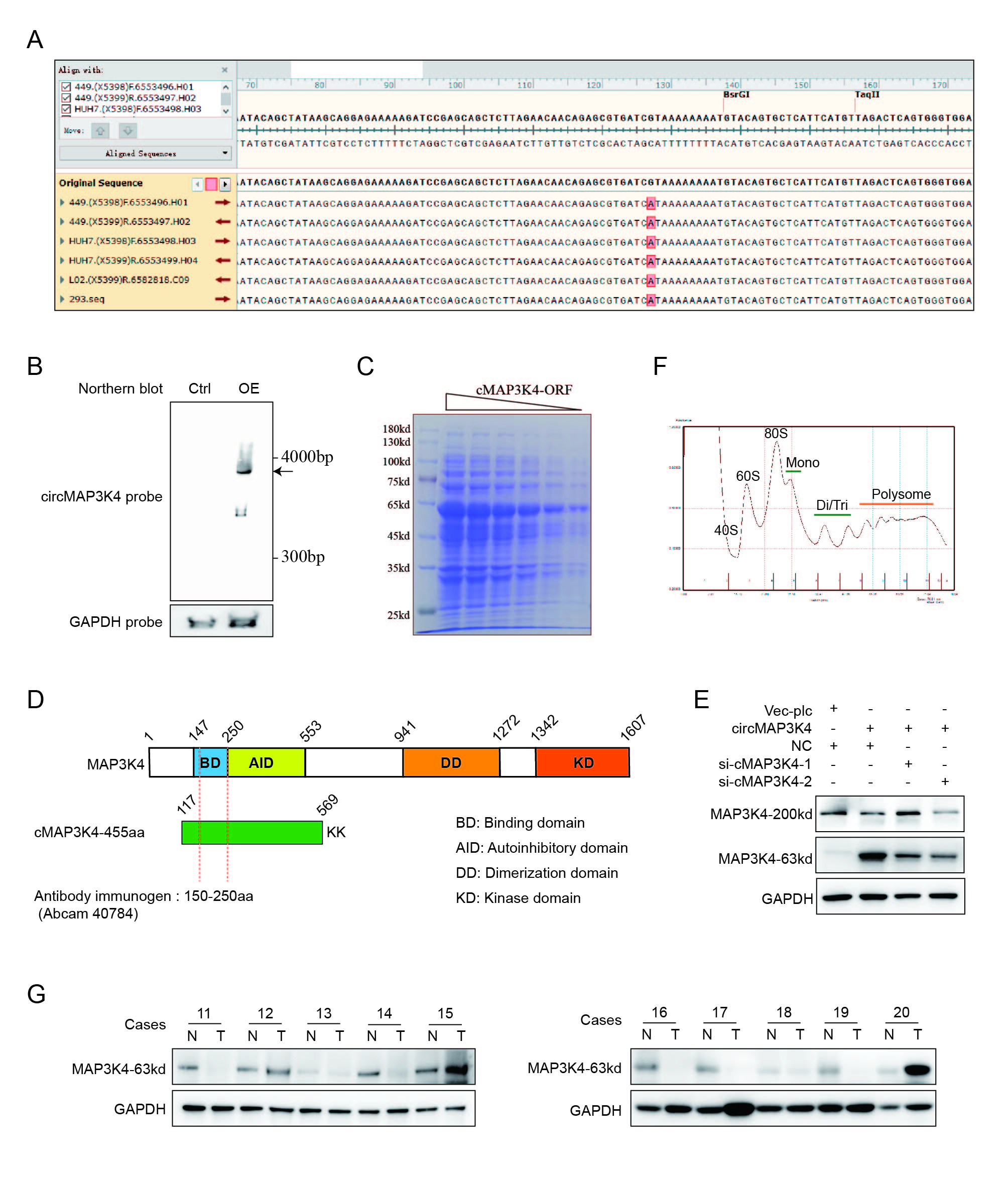


**Figure S2. CircMAP3K4 information and characteristics.** A. The circMAP3K4 SNP site. B. CircMAP3K4 expression detected by northern blot. C. Coomassie blue staining showing circMAP3K4-455aa expression in a prokaryotic system. D. Schematic illustrations of MAP3K4 protein and circMAP3K4-455aa. E. Western blot analysis for circMAP3K4-455aa expression after circMAP3K4 overexpression with or without specific circMAP3K4 siRNA. cMAP3K4, circMAP3K4. F. Ribosomes distribution of sucrose fractionation assays. G. Western blot analysis of circMAP3K4-455aa expression in additional 10 cases of HCC and the paired liver tissues.

**Figure S3**


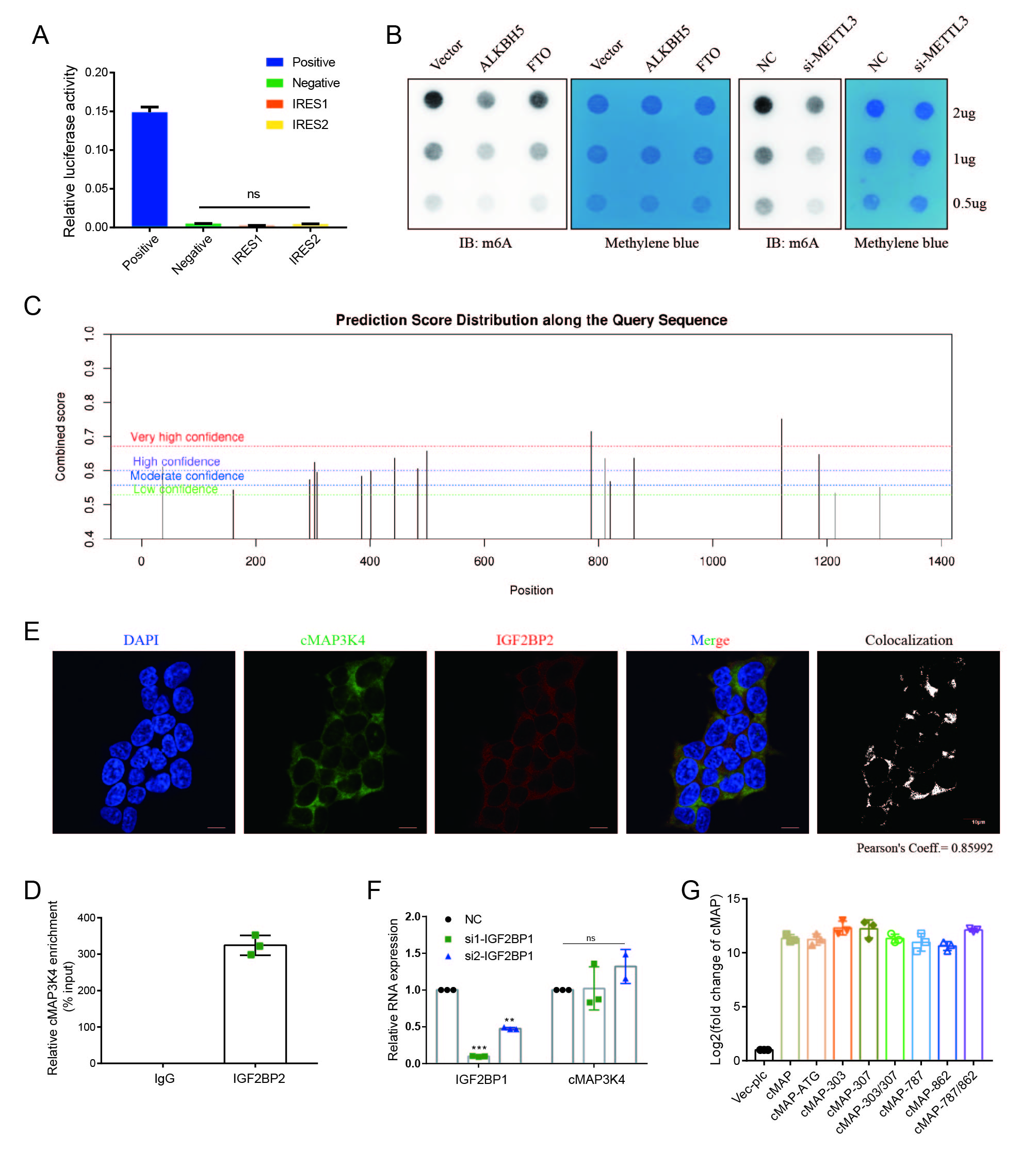
**Figure S3. m6A incorporation in circMAP3K4 translation.** A. CircMAP3K4 IRES-1 and IRES-2 identification and activity test using an IRES-luciferase reporter, inner with positive and negative controls. B. Dot blot showing m6A levels in indicated cells. C. The circMAP3K4 prediction score distribution. D, E. RIP (D) and IF-FISH (E) verified IGF2BP2 interaction with circMAP3K4. F. RT-qPCR analysis of circMAP3K4 in IGF2BP1 knockdown cells. G. RT-qPCR analysis of circMAP3K4 in each indicated group. IRESs: internal ribosome entry sites; cMAP3K4 or cMAP, circMAP3K4.

**Figure S4**


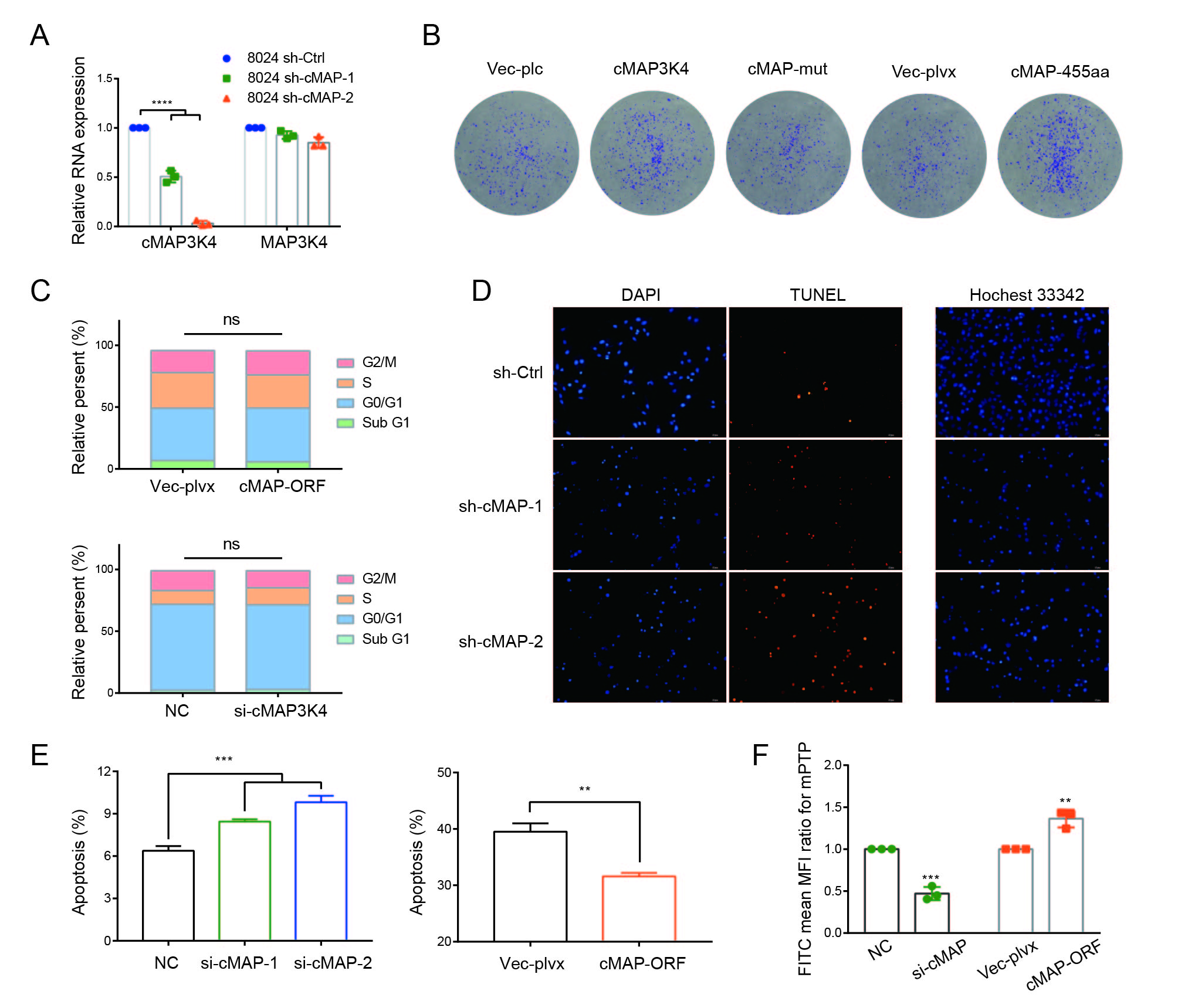
**Figure S4. The biological function and properties of circMAP3K4.** A. RT-qPCR of circMAP3K4 in the indicated cells. B. Representative clonogenic assay images from indicated groups. C. Cell cycle analysis examining the distribution of each phase in circMAP3K4-455aa and control cells. D. Representative TUNEL (left panel) and Hochest33342 staining (right panel) images showing apoptosis in each of the indicated groups. E. Annexin V/PI staining, an apoptosis indicator, in each group with treatment by oxaliplatin. F. Flow cytometry data bar chart with mPTP in the indicated cells. cMAP3K4 or cMAP, circMAP3K4; ns, not statistically significant.

**Figure S5**


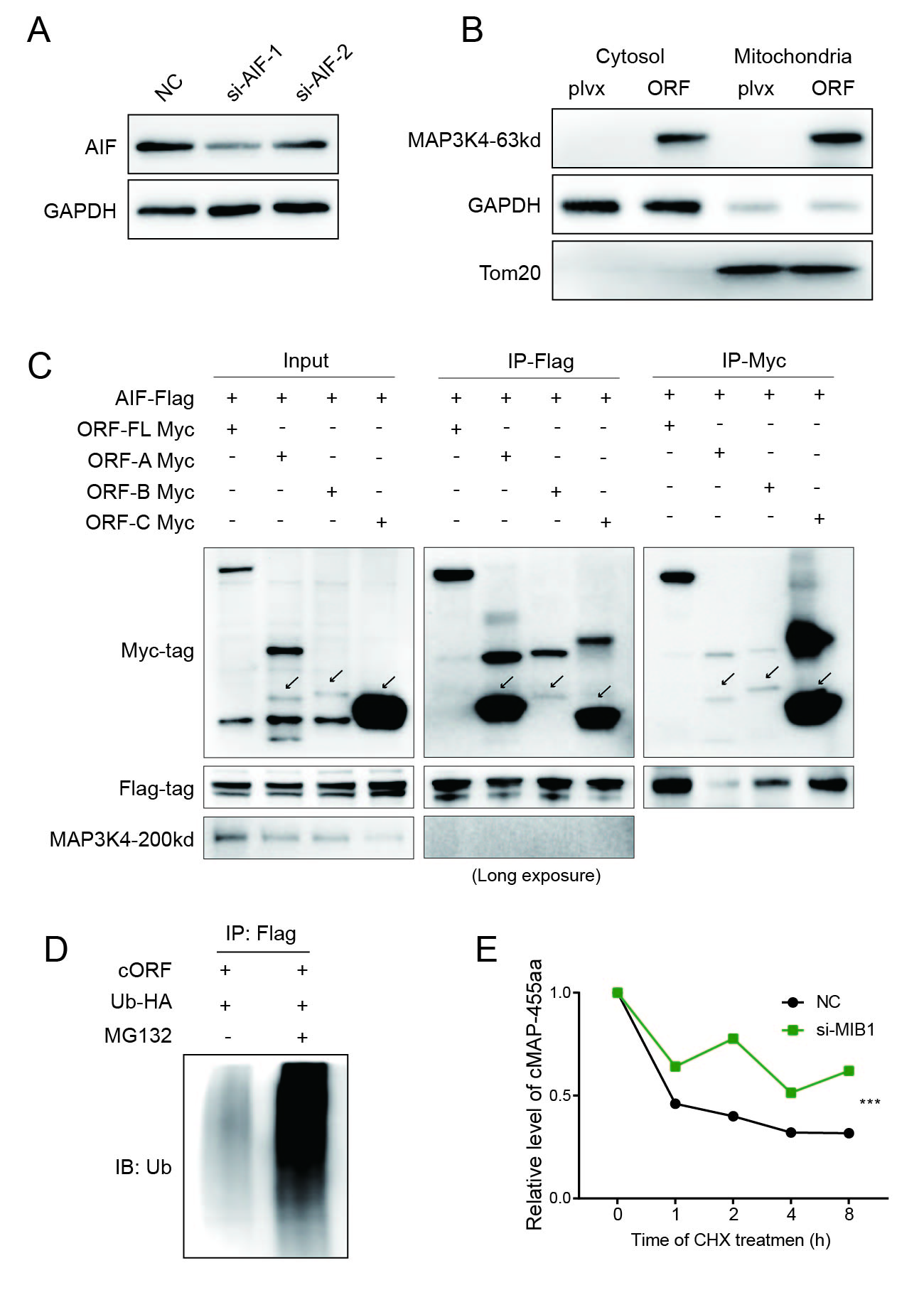


**Figure S5. CircMAP3K4 and its interacted proteins.** A. Western blot assay examining AIF knockdown efficiency. B. Western blot assay for the mitochondrial fractionation products. C. immunoblot analysis of immunoprecipitants after co-transfection with circMAP3K4-455aa mutant truncations and AIF. D. *In vivo* ubiquitylation assay assessing circMAP3K4-455aa ubiquitination. E. CircMAP3K4-455aa quantification at each indicated time after cycloheximide treatment.

**Supplementary tables**

**Table S1. Baseline characteristics of HCC patients.**

|  |  | No. (%) of patients | |  |
| --- | --- | --- | --- | --- |
| Characteristics | N = 112 (%) | CircMAP3K4 low expression  n = 52 | CircMAP3K4 high expression  n = 60 | *P* value |
| Gender |  |  |  | 0.775 |
| Male | 98 (87.5) | 45 (86.5%) | 53 (88.3%) |  |
| Female | 14 (12.5) | 7 (13.5%) | 7 (11.7%) |  |
| Age, years |  |  |  | 0.350 |
| ≤50 | 57 (50.9) | 24 (46.2) | 33 (55.0) |  |
| >50 | 55 (49.1) | 28 (53.8) | 27 (45.0) |  |
| AFP, ng/mL |  |  |  | 0.108 |
| ≤20 | 35 (31.5) | 20 (39.2) | 15 (25.0) |  |
| >20 | 76 (68.5) | 31 (60.8) | 45 (75.0) |  |
| ALT, U/L |  |  |  | 0.913 |
| ≤40 | 64 (57.1) | 30 (57.7) | 34 (56.7) |  |
| >40 | 48 (42.9) | 22 (42.3) | 26 (43.3) |  |
| AST, U/L |  |  |  | **0.009** |
| ≤40 | 52 (46.4) | 31 (59.6) | 21 (35.0) |  |
| >40 | 60 (53.6) | 21 (40.4) | 39 (65.0) |  |
| Differentiation |  |  |  | 0.136 |
| Well | 5 (4.5) | 3 (5.8) | 2 (3.3) |  |
| Moderate | 52 (46.4) | 29 (55.8) | 23 (38.3) |  |
| Poor | 53 (47.3) | 20 (38.5) | 33 (55.0) |  |
| Undifferentiated | 2 (1.8) | 0 (0) | 2 (3.3) |  |
| Tumor size, cm |  |  |  | 0.052 |
| ≤5 | 35 (31.3) | 21 (40.4) | 14 (23.3) |  |
| >5 | 77 (68.8) | 31 (59.6) | 46 (76.7) |  |
| Vascular invasion |  |  |  | 0.464 |
| Absence | 67 (59.8) | 33 (63.5) | 34 (56.7) |  |
| Presence | 45 (40.2) | 19 (36.5) | 26 (43.3) |  |
| Envelope |  |  |  | 0.279 |
| Absence | 65 (58.0) | 33 (63.5) | 32 (53.3) |  |
| Presence | 47 (42.0) | 19 (36.5) | 28 (46.7) |  |
| Liver cirrhosis |  |  |  | 0.206 |
| Absence | 61 (54.5) | 25 (48.1) | 36 (60.0) |  |
| Presence | 51 (45.5) | 27 (51.9) | 24 (40.0) |  |
| Necrosis |  |  |  | 0.093 |
| Absence | 82 (73.2) | 42 (80.8) | 40 (66.7) |  |
| Presence | 30 (26.8) | 10 (19.2) | 20 (33.3) |  |
| HBsAg |  |  |  | 0.884 |
| Negative | 4 (3.6) | 2 (3.8) | 2 (3.3) |  |
| Positive | 108 (96.4) | 50 (96.2) | 58 (96.7) |  |
| HBV DNA |  |  |  | 0.100 |
| ≤500 | 51 | 28 (53.8) | 23 (38.3) |  |
| >500 | 61 | 24 (46.2) | 37 (61.7) |  |
| Tumor multiplicity |  |  |  | 0.535 |
| Single | 89 (79.5) | 40 (76.9) | 49 (81.7) |  |
| Multiple | 23 (20.5) | 12 (23.1) | 11 (18.3) |  |

Abbreviations: AFP, alpha-fetoprotein; ALT, glutamic pyruvic transaminase; AST: aspartate aminotransferase; HBsAg, Hepatitis B surface antigen; HBV DNA, hepatitis B virus DNA.

p-values were calculated with the two-sided log-rank test.

**Table S2. Multivariable analyses of risk factors for patients’ survival.**

| Characteristics* | Overall survival | |  | Disease-free survival | |
| --- | --- | --- | --- | --- | --- |
|  | HR (95% CI) | *P* value |  | HR (95% CI) | *P* value |
| CircMAP3K4 (low *vs* high) | 1.662 (1.083-2.549) | 0.020 |  | 1.733 (1.130-2.656) | 0.012 |
| Gender (male *vs* female) | 0.626 (0.291-1.347) | 0.231 |  | 0.827 (0.426-1.606) | 0.575 |
| Age (≤50 *vs* >50 years) | 0.800 (0.514-1.246) | 0.323 |  | 0.791 (0.504-1.241) | 0.307 |
| AFP (≤20 *vs* >20 ng/mL) | 1.378 (0.857-2.217) | 0.186 |  | 1.550 (0.983-2.445) | 0.060 |
| ALT (≤40 *vs* >40 U/L) | 1.284 (0.806-2.045) | 0.293 |  | 1.616 (1.011-2.581) | 0.045 |
| AST (≤40 *vs* >40 U/L) | 1.479 (0.812-2.692) | 0.201 |  | 2.059 (1.062-3.993) | 0.033 |
| HBV DNA (≤500 *vs* >500 copies/L) | 0.949 (0.589-1.530) | 0.830 |  | 1.061 (0.675-1.668) | 0.798 |
| Differentiation (Well-moderate *vs* poor-undifferentiated) | 1.216 (0.757-1.952) | 0.418 |  | 1.357 (0.935-1.969) | 0.108 |
| Tumor size (>5 *vs* ≤5 cm) | 1.884 (1.114-3.185) | 0.018 |  | 1.681 (1.082-2.612) | 0.022 |
| Vascular invasion (absence *vs* presence) | 1.430 (0.910-2.246) | 0.120 |  | 1.736 (1.144-2.634) | 0.010 |
| Envelope (absence *vs* presence) | 1.693 (0.979-2.927) | 0.059 |  | 1.048 (0.673-1.632) | 0.836 |
| Liver cirrhosis (absence *vs* presence) | 1.007 (0.652-1.556) | 0.973 |  | 1.020 (0.660-1.577) | 0.928 |
| Necrosis (absence *vs* presence) | 1.370 (0.832-2.255) | 0.217 |  | 1.085 (0.648-1.819) | 0.756 |
| Tumor multiplicity (single *vs* multiple) | 2.203 (1.275-3.807) | 0.005 |  | 1.851 (1.081-3.171) | 0.025 |

*Due to small numbers of cases in negative group of HBsAg variable, HBsAg was not included in multivariable analysis.

Abbreviations: AFP, alpha-fetoprotein; ALT, glutamic pyruvic transaminase; AST: aspartate aminotransferase; HBV DNA, hepatitis B virus DNA; HR, hazard ratio; CI, confidence interval.

p-values were calculated with the two-sided log-rank test.

**Table S3. Referenced primers and probes.**

| circMAP3K4-F primer | 5’-CGATCGTCCAGTGGAGAAAAA-3’ |
| --- | --- |
| circMAP3K4-R primer | 5’-GGCTGAGGTAAGGCTTATTG-3’ |
| MAP3K4-F primer | 5’-ACCGAGTCAGAACCCGAGTG-3’ |
| MAP3K4-R primer | 5’-ATCGGAGAAGTCTTCTAGATCAG-3’ |
| GAPDH-F primer | 5’-TGCACCACCAACTGCTTAGC-3’ |
| GAPDH-R primer | 5’-GGCATGGACTGTGGTCATGAG-3’ |
| U3-F primer | 5’- GAGCACCGAAAACCACGAG-3’ |
| U3-R primer | 5’- CCGCGTTCTCTCCCTCTC-3’ |
| 18S-F primer | 5’- GCGGCTTTGGTGACTCTAGA -3’ |
| 18S-R primer | 5’- TGCCTTCCTTGGATGTGGTA -3’ |
| IGF2BP1-F primer | 5’-GCGGCCAGTTCTTGGTCAA-3’ |
| IGF2BP1-R primer | 5’-TTGGGCACCGAATGTTCAATC-3’ |
| IGF2BP2-F primer | 5’-AGCTAAGCGGGCATCAGTTTG-3’ |
| IGF2BP2-R primer | 5’-CCGCAGCGGGAAATCAATCT-3’ |
| circMAP3K4 probe | 5’-AUUUGGUGCAUUCAUUUUUUCUCCACUGGACGAUCGUUCUUUACCAAUG-biotin-3’ |
| NC probe | 5’-UUGUACUACACAAAAGUACUG-biotin-3’ |
| GAPDH probe | 5′-UAUCCACUUUACCAGAGUUAAAAGCAGCCCUGGUGACCAGGCGCCCAAUACGACCAAA-biotin-3′ |
